# Epidemiology of Human Papillomavirus in the Middle East and North Africa: A Review of Genotypic Prevalence, Vaccination Challenges, and Non-Cervical Clinical Outcomes

**DOI:** 10.3390/diseases14080302

**Published:** 2026-08-19

**Authors:** Maedeh Mirasheh, Zahra Shahabinia, Afrooz Mazidimoradi, Leila Allahqoli, Hamid Salehiniya, Do-Youn Lee

**Affiliations:** 1 Department of Midwifery and Reproductive Health, School of Nursing and Midwifery, Tehran University of Medical Sciences, Tehran 1419733171, Iran; m_mirasheh@razi.tums.ac.ir; 2 Geriatric Health Research Center, Birjand University of Medical Sciences, Birjand 9717853577, Iran; 3 Health Assistant Department, Shiraz University of Medical Sciences, Shiraz 7193711351, Iran; 4 Faculty of Health Sciences, Cyprus International University, Nicosia 99258, Cyprus; 5 Department of Epidemiology and Biostatistics, School of Health, Social Determinants of Health Research Center, Birjand University of Medical Sciences, Birjand 9717853577, Iran; 6 College of General Education, Kookmin University, Seoul 02707, Republic of Korea

**Keywords:** human papillomavirus, cervical cancer, Middle East, North Africa, epidemiology

## Abstract

Background: Human papillomavirus (HPV) is the most common sexually transmitted infection and the primary etiological agent of cervical cancer. Despite the high global disease burden, the Middle East and North Africa (MENA) region faces substantial challenges in HPV prevention and control. This narrative review aimed to synthesize existing evidence on the prevalence, genotypic distribution, awareness, vaccination status, clinical outcomes, and the role of HPV in non-cervical malignancies in the MENA region. Methods: PubMed, Web of Science, Scopus, and Google Scholar were searched, and 122 studies were included. Findings were synthesized qualitatively and descriptively. Cohort studies, systematic reviews, and meta-analyses were prioritized to support inferences about associations and potential causal relationships. Results: HPV prevalence in the general female population of the MENA region ranged from 4.7% to 53.3%, exceeding 90% in high-risk groups and patients with malignancies. The most common genotypes were HPV16 and HPV18; however, distribution patterns varied across populations. Key risk factors included younger age, multiple sexual partners, early age at first sexual intercourse, smoking, and alcohol consumption. General awareness was low to moderate, and vaccination coverage was very low in most countries. Barriers included lack of knowledge, fear of side effects, religious concerns, and social stigma. Evidence also indicates an association between HPV and non-cervical malignancies (head and neck, esophageal, gastric, breast, and thyroid cancers), with HPV DNA detected in tumor tissues. Conclusions: HPV poses a significant public health challenge in the MENA region, characterized by high prevalence, genotypic diversity, limited knowledge and awareness, and unsatisfactory vaccination coverage. Expanding screening and vaccination through financial support, culturally and religiously appropriate education, and longitudinal studies to monitor genotypic changes and associations with other malignancies are essential measures to reduce the burden of HPV-related diseases in the region.

## 1. Introduction

Human papillomavirus (HPV) is the most common sexually transmitted infection worldwide [1,2]. With more than 200 identified types, it is recognized as the primary cause of cervical cancer [1,3] and is associated with other malignancies, including those of the head and neck, breast, colorectal, esophageal, gastric, and thyroid cancers [4,5,6,7,8,9,10,11,12]. According to statistics reported by the International Agency for Research on Cancer (IARC), cervical cancer ranks as the fourth most common cancer in terms of both incidence and mortality, with approximately 660,000 new cases and 350,000 deaths occurring globally each year [13,14]. The incidence rate of cervical cancer has been reported as 3.4 per 100,000 in the Greater Middle East and 6.4 per 100,000 in North Africa [15,16].

Culture and religious beliefs in the MENA region are relatively similar, and sexual behaviors tend to be more conservative than those in Western societies. Consequently, the prevalence of sexually transmitted infections in this region was previously estimated to be lower than in other parts of the world. However, in recent years, owing to rapid changes in lifestyle—particularly among the younger generation—and a shift towards greater behavioral freedom, the prevalence of sexually transmitted infections, including HPV, has been increasing in these populations [17,18].

In addition to cervical cancer, evidence supports HPV’s role in other malignancies in this region. Cancers of the head and neck, esophagus, and stomach, as well as malignancies of the breast and thyroid, have been reported, with HPV DNA detected in tumor tissue samples from these cancers [5,6,7,8,9,10,11,19].

Despite this, the MENA region faces fundamental challenges in HPV prevention and control. Most countries in this region lack national programs for HPV screening and vaccination. Furthermore, the absence of accurate cancer registries in most of these countries complicates precise assessment of the disease burden [20,21,22]. Numerous studies have shown that general awareness regarding HPV infection and its vaccine is low to moderate, with barriers to vaccine acceptance including lack of knowledge, concerns about side effects, high costs, and social stigma [23,24,25,26,27,28,29,30].

Given the increasing trend of HPV prevalence in MENA countries, the diverse distribution of viral genotypes across different populations, the lack of public awareness, low vaccination coverage, and the virus’s association with a wide spectrum of malignancies, few studies have simultaneously investigated these aspects. The specific objectives of this narrative review are: (1) to investigate the prevalence and genotype distribution pattern of HPV, (2) to identify associated risk factors, (3) to assess the level of public and professional awareness, (4) to assess the status of vaccination and its barriers, and (5) to review the role of HPV in non-cervical malignancies. Therefore, conducting this review is essential. Furthermore, the findings may facilitate the formulation of culturally tailored screening and vaccination policies, which could help reduce the burden of HPV-related diseases in the region.

## 2. Materials and Methods

### 2.1. Study Design

This study was conducted as a narrative review aimed at comprehensively examining the epidemiology, genotypic distribution, risk factors, public and professional awareness, vaccination status, and the role of HPV in non-cervical malignancies in the Middle East and North Africa (MENA) region.

### 2.2. Search Strategy

We searched the PubMed, Web of Science, and Scopus databases, as well as the Google Scholar search engine, on 20 May 2026 for articles published between January 2016 and May 2026. Only articles published in English were included in the study. The search strategy combined the keywords “human papillomavirus,” “HPV,” “Epidemiology,” “Prevalence,” “Vaccination,” “cancer,” “Middle East,” “North Africa,” and “MENA.”

### 2.3. Eligibility Criteria

#### 2.3.1. Inclusion Criteria

Inclusion criteria comprised articles investigating prevalence and genotypic distribution, co-infections, risk factors, general and specialized awareness and attitudes, vaccination status, viral genetic diversity, and HPV in other malignancies and specific populations in the MENA region.

Geographic scope: MENA region was defined as 21 countries according to the World Bank classification [31]. However, Turkey and Mauritania were also included in our operational definition because they are frequently mentioned in health studies related to the Middle East and North Africa and share common geographical and cultural links with the region. Therefore, the final geographical scope encompassed 23 countries.

#### 2.3.2. Exclusion Criteria

Studies that did not provide clinical and epidemiological data for populations in the region were excluded.

### 2.4. Study Selection, Data Extraction, and Synthesis

After removing duplicate articles, titles and abstracts were reviewed, followed by an evaluation of full-text articles. The studied populations included women from the general population, patients with malignancies, men, children, students, and healthcare professionals. Biological samples examined included Pap smear and biopsy samples from the cervix, oropharyngeal samples, tumor tissues from the breast, stomach, esophagus, and thyroid, semen samples, and blood samples.

Data extraction was performed qualitatively, and findings related to HPV prevalence in various populations and associated factors were synthesized.

Data synthesis was also conducted narratively. Owing to the nature of the narrative review and the high heterogeneity of the studied populations, a meta-analysis was not performed, and findings were reported descriptively, highlighting similarities and contradictions among studies. For conclusions regarding populations and causal relationships, priority was given to cohort studies, systematic reviews, and high-quality meta-analyses. Findings from studies with small sample sizes and specific populations were used to describe regional patterns more precisely. As an inherent limitation of the narrative review design, we did not conduct formal quality assessments of individual studies using standard tools; however, we prioritized rigorous methodologies when drawing conclusions.

## 3. Results

In this narrative review, of the 2332 studies identified in the initial search, 122 studies were selected for the final analysis after removing duplicate articles (*n* = 461) and screening the titles, abstracts, and full texts according to the inclusion criteria. Given the significant heterogeneity of the studies in terms of methodology, populations studied (including reproductive-age women, high-risk groups, men, and children), diverse biological samples (cervical smears and biopsies, oropharyngeal samples, breast, stomach, esophagus, and thyroid tumor tissues, semen, and blood), and different research objectives, the findings were organized narratively into main themes comprising epidemiological patterns and genotype distribution, demographic and behavioral risk factors, public and professional awareness levels and barriers to vaccination, cytological and histopathological correlations, viral genetic diversity and molecular consequences, the role of HPV in non-cervical malignancies (head and neck, esophagus, stomach, breast, and thyroid), and epidemiology in specific populations (men, children, and high-risk groups). This approach provides a comprehensive picture of the available evidence on HPV epidemiology in the Middle East and North Africa region. The characteristics of the articles included in this review are presented in the Supplementary Table S1.

### 3.1. Epidemiology and Genotype Distribution of HPV

#### 3.1.1. Epidemiological Patterns and Genotypic Distribution

The prevalence of HPV in the MENA region exhibits significant heterogeneity, influenced by geographic location, diagnostic methods, and study populations. In the general female population, reported rates vary widely from 4.7% to 53.3% [32,33,34,35,36,37,38,39,40,41,42,43,44,45,46,47,48,49,50,51,52,53,54,55,56,57,58,59]. This broad range reflects methodological differences; for instance, studies utilizing highly sensitive PCR techniques often report higher prevalence compared with those using cytology-based screening. Generally, prevalence is higher in Gulf Cooperation Council (GCC) countries (e.g., UAE at 42%, Saudi Arabia 6.9–15.1%) and North African nations (e.g., Egypt 13.5–37.8%, Morocco 4.9%) [32,33,34,35,36,37,38,39,40,41,42,43,44,45,46,47,48,49,50,51,52,53,54,55,56,57,58,59].

In contrast, prevalence is markedly elevated in high-risk groups and clinical populations. Among women with cervical lesions, positivity rates exceed 90% in cases of invasive cancer and high-grade intraepithelial neoplasia (CIN2/3) [60,61,62,63,64,65]. For example, studies in Mauritania and Iran reported HPV positivity rates of 94% and 93.6% among women with high-grade lesions, respectively [60,62]. Similarly, HIV-positive women showed significantly higher prevalence (up to 76.25%) compared with the general population [61].

#### 3.1.2. Predominant Genotypic Patterns: A Comparison of High-Risk and Low-Risk Types

As summarized in Table 1, genotype distributions differed between the general female population and high-risk/clinical populations. HPV16 and HPV18 remain the most predominant high-risk genotypes across the region, consistent with global trends [35,36,39,40,46,50,53,59,63,64,66,67,68,69,70,71,72,73,74]. However, regional variations exist; genotypes such as HPV33, HPV31, HPV56, and HPV52 have shown higher relative frequencies in certain populations, particularly in Iran and Iraq [36,43,61,63,75,76,77]. Regarding low-risk types, HPV6 and HPV11 are consistently the most common, and are associated primarily with genital warts [41,44,59,64,74,76,78].

Notably, the distribution of genotypes correlates with lesion severity. HPV16 prevalence increases with the progression from benign lesions to invasive carcinoma, reaching up to 92% in invasive cancer cases, whereas other genotypes like HPV33 are more frequently detected in lower-grade lesions [63]. Furthermore, viral load tends to increase with disease severity, being significantly higher in invasive cancers than in pre-malignant CIN2/3 lesions [60]. Generally, high-risk and low-risk HPV genotypes show diverse distribution patterns in different populations, but HPV16 and HPV18 dominated among high-risk genotypes, and HPV6 and HPV11 among low-risk genotypes in most studies. However, these patterns varied in some regions depending on lesion severity.

#### 3.1.3. Co-Infections and Multiple Infections

Co-infection with multiple HPV genotypes is a notable phenomenon in the MENA region, with prevalence ranging from 0.5% to 53.8% depending on the detection method and population studied [33,36,42,47,55,56,65,71,74,79,89]. Mixed infections involving two or more genotypes were observed in approximately half of the HPV-positive cases in some cohorts [56,90]. HPV16 was the most frequent genotype found in multiple infections, often co-occurring with HPV31, 35, and 39 [71].

Interestingly, the relationship between the number of infecting genotypes and lesion severity appears complex. Some studies suggest that single high-risk infections are more common in advanced malignancies, while multiple infections are more prevalent in normal cytology or low-grade lesions (LSIL) [90]. Conversely, other research indicates no significant association between the number of genotypes and cervical pathology severity [65].

Beyond multiple HPV types, concurrent infections with other sexually transmitted infections (STIs) are common. HPV-positive individuals show significantly higher rates of co-infection with Mycoplasma genitalium, Chlamydia trachomatis, and Herpes Simplex Virus (HSV) [79,89]. These co-infections may synergistically increase the risk of cervical dysplasia and progression to cancer [79,89]. Additionally, a strong association exists between HPV and HIV infection, with HIV-positive women exhibiting higher HPV prevalence and persistence [79,89].

Overall, concurrent infections with HPV are influenced by various factors and are associated with different clinical outcomes.

### 3.2. Risk Factors, Related Behaviors, and Impact on Quality of Life

#### 3.2.1. Demographic and Behavioral Risk Factors

HPV infection is influenced by various demographic and behavioral factors. Age is the most significant demographic determinant, with HPV prevalence generally higher in younger women (particularly those aged 20–30) and declining with advancing age [45,47,50,56]. However, this trend is not universal, as some studies report increased prevalence with age [84]. Ethnicity and nationality also influence risk, with non-Saudi women showing higher rates in Saudi Arabia [52]. Gender effects are inconsistent; while one study found HPV16 more common in men, others reported higher high-risk HPV prevalence in women [74,91]. Conversely, education level, place of residence, and marital status showed no significant correlation with HPV infection across most studies [33,51,59,78].

Smoking and alcohol consumption are significant behavioral risk factors for HPV infection and disease progression. Smoking rates were notably higher among HPV-positive individuals compared with controls, with prevalence increasing alongside the severity of cervical lesions (from CIN1 to invasive cancer) [62,64,72]. In a cross-sectional study of women undergoing cervical evaluation, the rate of smokers among HPV-positive individuals was 11.7%, while it was zero among HPV-negative individuals [64]. Alcohol consumption also showed a positive correlation with HPV positivity [62].

**Figure 1:**
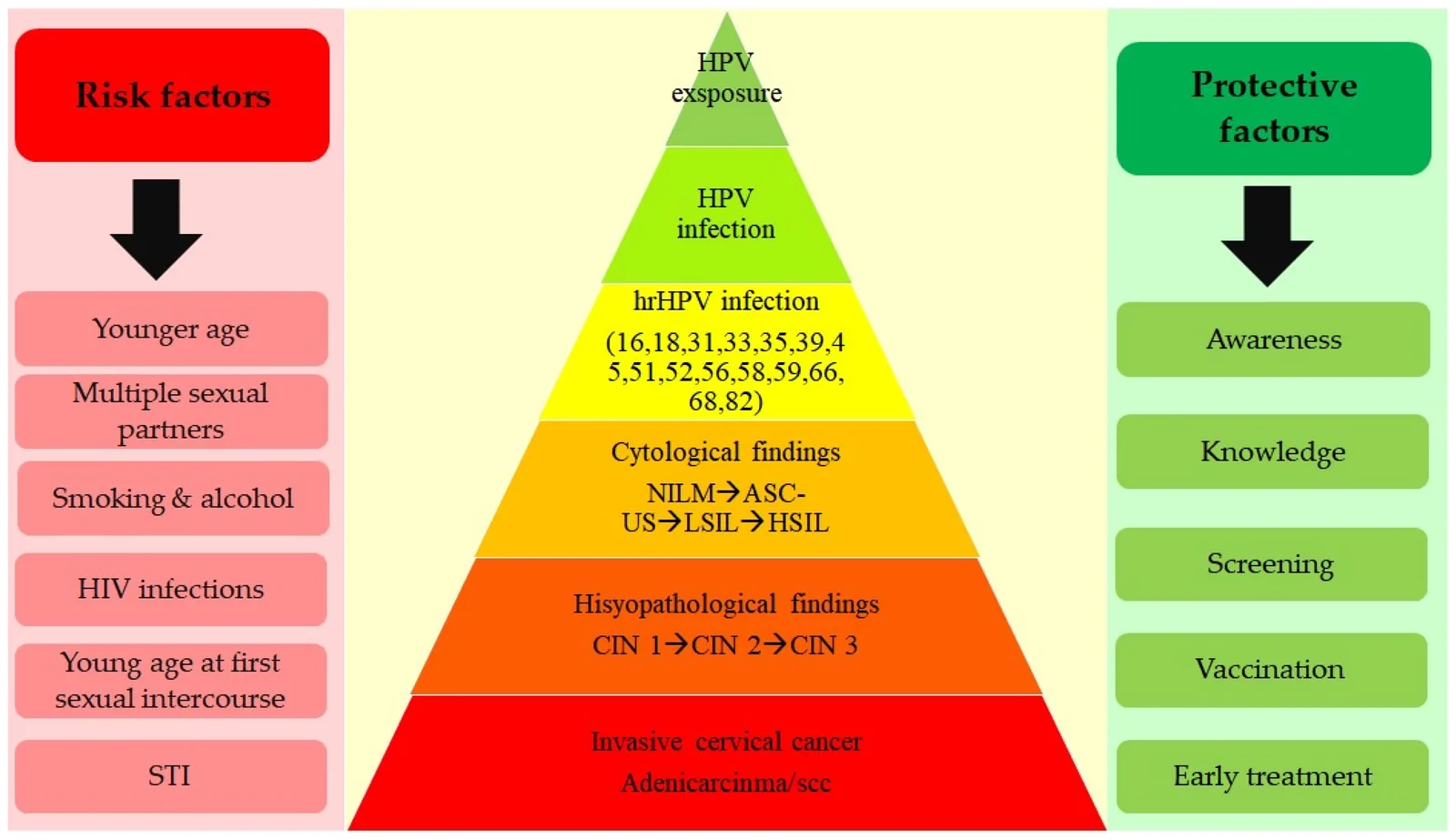
Stages of disease progression, risk factors, and protective factors of HPV.

Regarding sexual behavior, having multiple sexual partners, an early age at first intercourse, and a history of sexually transmitted diseases (STDs) significantly increase HPV risk. Studies indicate that HPV-positive individuals have higher rates of multiple partners and concurrent STDs compared with uninfected individuals [64]. Furthermore, younger age at first sexual intercourse is associated with more severe cervical abnormalities (e.g., lower mean age in CIN3 vs. normal groups) [68]. Additionally, an increased number of marriages was significantly linked to higher infection risk, with each additional marriage raising the odds by 107% [55]. These factors collectively contribute to disease progression, whereas vaccination and screening remain key protective measures (Figure 1).

#### 3.2.2. Impact of HPV on Quality of Life

HPV infection, particularly when manifesting as genital warts, significantly impairs women’s quality of life and sexual function. Infected individuals report lower overall quality of life scores compared with uninfected controls [34]. The presence of genital warts is associated with increased sexual distress, shame, marital difficulties, and relationship disorders [92,93]. Furthermore, HPV-positive women frequently experience psychological burdens, including shock, shame, fear of cancer, social isolation, concern about infertility, and concern about transmission to spouses [83]. These findings highlight the substantial psychosocial impact of HPV beyond its physical symptoms.

### 3.3. Public and Healthcare Professionals Awareness, Vaccination Status, and Barriers

#### 3.3.1. General Awareness and Knowledge Gaps

Multiple studies indicate that general awareness of HPV infection and its associated risks in the MENA region is generally low to moderate. In Saudi Arabia, significant portions of the adult population reported poor knowledge regarding HPV transmission and carcinogenicity, with some studies showing up to 67.3% having poor knowledge [23,24]. Similarly, in Egypt, 86.5% of participants lacked sufficient knowledge [94], while in Turkey, mean awareness scores were significantly below expected levels (4.39/10) [25]. Misconceptions are prevalent; for instance, beliefs that HPV is treatable with antibiotics or transmitted solely through penetrative intercourse persist among the public [24,94,95]. Demographic analyses consistently show that higher education levels, female gender, and younger age are associated with better awareness scores [25,29,94,96,97,98,99,100,101].

#### 3.3.2. Awareness Among Healthcare Professionals

While healthcare professionals generally demonstrate higher knowledge levels than the general public, significant gaps remain across different disciplines. Studies report high awareness among doctors (90.4%) and nurses (86.7%), but substantially lower rates among dentists (72%) and pharmacists (31.2%) [102]. Among students, clinical trainees exhibit greater awareness than pre-clinical peers, and medical students often have higher knowledge scores than those in other fields [98,103,104]. However, despite adequate theoretical knowledge, practical application remains limited; for example, only 16.6% of Iranian medical students had received the vaccine [105], and nursing students answered only 36.6% of the awareness questions correctly [106].

#### 3.3.3. Vaccination Coverage and Trends

HPV vaccination coverage in the MENA region is predominantly low, reflecting the absence of widespread national immunization programs. In Iran, vaccination rates are critically low, ranging from 0.1% to less than 1% in various studies [32,97,107]. Coverage in GCC countries varies but remains modest: rates ranged from 2.7% in Kuwait to 18.9% in the UAE, with an overall average of 8.8% among adults aged 18–39 [26,108,109,110]. Notably, non-GCC citizens in these regions showed significantly higher uptake (21.4%) compared with locals (5.5%) [26]. Conversely, countries with established national programs, such as Turkmenistan, Uzbekistan, and Armenia, achieved high coverage rates (33.7–99.3%) by age 15 [111]. This stark contrast highlights the critical role of government support and programmatic inclusion in driving vaccination uptake. Even among high-risk groups, such as women already diagnosed with HPV, uptake remains low (approximately 3.1%) [27].

#### 3.3.4. Barriers to Vaccination

The low vaccination rates are driven by a complex interplay of barriers, primarily categorized into awareness deficits, safety concerns, socio-cultural stigma, and economic factors.

1. Lack of Awareness: Insufficient knowledge about the vaccine’s existence and benefits is a primary deterrent. In GCC countries, 53.6% of unvaccinated individuals cited lack of awareness as the main barrier [26], a figure rising to 57.3% in Saudi Arabia [27] and affecting 79% of participants in Iran who lacked sufficient knowledge about the vaccine itself [97].

2. Safety Concerns and Stigma: Fear of side effects is a major concern, cited by 21.9–30.5% of unvaccinated individuals in Saudi Arabia and 29.4% in Iran regarding immune efficacy [27,28,112]. Furthermore, deep-seated socio-cultural stigmas link vaccination to sexual promiscuity, particularly among parents in Kuwait and Saudi Arabia, hindering acceptance [30,113].

3. Economic Constraints: Cost is frequently identified as a significant barrier. In Saudi Arabia, 22.5% cited cost as the main obstacle, with 93.9% believing the Ministry of Health should cover it [28]. Similar trends were observed in Kuwait [114] and among patients with anogenital warts in Iran, where 60% mentioned cost [96]. A study in Iran further confirmed that high costs outweigh perceived efficacy in preventing disease [115].

In summary, while awareness among healthcare providers is relatively high, this does not translate into high vaccination rates owing to systemic barriers including cost, cultural stigma, and persistent misconceptions within the general population.

### 3.4. Cytological and Histopathological Correlations with Genotypes

High-risk HPV genotypes are associated with the development and progression of cervical cytological and histopathological lesions [41,47,58,62,64,68,72]; specifically, genotype is directly correlated with the severity of cervical abnormalities [47,58,64,68,85,116].

Increased abnormal findings in cytology were associated with HPV positivity. In the HPV mRNA test, the rate of positive cases in normal cytology was 3.5%, increasing to 33.5%, 64.4%, and 100% in ASC-US, LSIL, and HSIL, respectively [116]. Similarly, in the HPV DNA test, it reached 6.6% in NILM, 50% in ASC-US, 70.8% in LSIL, and 85.7% in HSIL [116]. Also, in another study, a significant and positive correlation was observed between HPV-positive status and cellular changes in Pap smears (ASC-US, LSIL, and HSIL) [78]. In HPV-positive women, 39% were ASC-US, 27% LSIL, 11% ASC-H, and 5.4% HSIL [58]. Also, in a review of 3510 women, high-risk HPV was positive in 42.1% of abnormal cytology and 7.9% of normal cytology, with the highest rates observed in LSIL (77.4%) and HSIL (75%) [47].

Regarding genotype types, HPV16 had higher prevalence in LSIL at 26% and ASC-US at 13–33% [64]. In HSIL, HPV31 was 26%, and in ASC-US and LSIL, HPV16 had prevalences of 13% and 26%, respectively, while HPV-18, HPV-52, HPV-53, and HPV-58 were less common [65]. Also, in another review, HPV16 and HPV18 were common genotypes in NILM, ASC-US, LSIL, HSIL, and SCC cytologies, but in adenocarcinoma, HPV16 and HPV45 were more frequently seen [64]. Furthermore, in one review, HPV16 was found in adenocarcinoma, and HPV18, 35, 52, and 61 in squamous cell carcinoma [73].

In biopsies, HPV16 was reported in 47.8% of CIN1, 23.8% of CIN2/3, and 28.4% of normal lesions [62]. In other studies, HPV16 increased with severity, with the lowest rate in CIN1 and an upward trend reaching about 90% in invasive cancer [58,64,68]. Conversely, HPV18 decreased in more severe stages, from 16.7% in CIN1 to 11.4% in CIN3 [68]. In SCC, the single HPV16 genotype was in 63.15%, and when accompanied by other genotypes, it dropped to 23.15% [117] (Table 2 and Table 3).

**Table 2:** Cytology Findings with Most Common HPV Genotypes.

Finding	Prevalent HPV Genotypes	Reference
NILM	HPV16 HPV18 HPV6 HPV52 HPV53 HPV68	[34,41,47,54,56,62,64,65,72,74,79,116]
ASC-US	HPV16 HPV31 HPV53 HPV18	[34,47,53,56,58,61,62,64,65,72,78,80,85,116,118]
LSIL	HPV16 HPV58 HPV53	[34,47,56,64,65,116]
HSIL	HPV16 HPV18 HPV31 HPV52 HPV39	[34,47,56,64,65,116]

**Table 3:** Histopathology Findings with Most Common HPV Genotypes.

Finding	Prevalent HPV Genotypes	Reference
Normal	HPV16 HPV18 HPV6	[62,64]
CIN1	HPV16 HPV18 HPV31 HPV53 HPV58	[49,54,58,62,64,68,72]
CIN2/3	HPV16 HPV18 HPV31 HPV58	[49,53,54,58,62,64,68,72,116,118]
SCC	HPV16 HPV18 HPV6 HPV11	[49,53,54,64,68,72,117,118]
Adenocarcinoma	HPV16 HPV18 HPV45	[49,53,54,64,73,118]

### 3.5. Viral Genetic Diversity and Molecular Implications

#### 3.5.1. Viral Genetic Diversity and Clinical Implications

Genetic diversity of HPV affects carcinogenesis and progression of cervical lesions by increasing the expression of E6/E7 oncogenes. Phylogenetic analyses reveal distinct lineage distributions across high-risk genotypes. For HPV16, Lineage D is predominant (66.4–79.2%), followed by Lineage A; notably, Lineage D shows a higher rate of viral integration into the host genome during progression to invasive cancer compared with European variants, which are more common in low-grade lesions (LSIL) [90,119,120]. Specific mutations, such as L83V in the E6 gene of HPV16, are highly prevalent and may influence oncogenic potential [90,119]. Similarly, HPV18 exhibits a uniform distribution of Type A lineages, with the C7486T mutation creating binding sites for transcription factors like c-Fos, thereby enhancing viral gene expression in most samples [121,122]. Other genotypes also show specific dominance patterns: HPV58 is predominantly Lineage B (91.5%) with frequent E6 mutations (e.g., E32Q), while HPV33, HPV35, and HPV52 are largely associated with Lineage A variants [75,123,124]. Despite these detailed molecular characterizations, current evidence does not consistently demonstrate a strong correlation between specific viral lineages or point mutations and the severity of cervical intraepithelial neoplasia (CIN) or clinical outcomes [122]. Therefore, while genetic diversity provides insights into viral evolution and potential pathogenic mechanisms, its direct utility in guiding clinical management or predicting disease prognosis in the MENA region remains limited due to heterogeneity in study findings.

#### 3.5.2. Molecular Changes

In HPV-positive groups, the expression of cell cycle genes, including CCNE1 and CDK2, was significantly higher than in non-infected groups [62]. Also, MALAT1 (9.75-fold), PI3K (24.59-fold), H19 (7.1-fold), and LINC00460 (1.15-fold) were increased in HPV-positive samples and positively correlated with oncoproteins E6/E7 [125]. The E6 protein is expressed in 50% of invasive cancer cells and 30% of pre-malignant lesion cells. This expression pattern indicates cellular senescence (OIS) in early lesions, but in advanced stages, cells escape this process and become malignant [126].

### 3.6. HPV Epidemiology in Specific Populations (Men, Children, and High-Risk Groups)

#### 3.6.1. HPV in Men

HPV infection in men has varying prevalence depending on the sample type and studied population. In different studies, HPV prevalence in men ranged from 13% to 76% [37,38,40,43,76]. In a study of 177 men, 13% were HPV positive, with 40% having high-risk genotypes [37]. Also, in another study, prevalence was 27.5% [40]. In some studies, HPV prevalence in men was higher [43,76]; however, in others, there was no significant difference by gender [38].

Regarding genotype distribution, in a study on HPV-positive men, 100% of men were positive for HPV6 [35]. Also, in HPV-positive men, low-risk genotypes were observed in 91.7% and high-risk in 25% [36]. In men with cutaneous warts, 46.7% were positive for HPV27b and 26.3% for HPV57c [127]. Furthermore, sexual function and quality of life in men significantly decreased if they contracted genital warts [128]. Regarding impact on fertility, using the Mann–Whitney U test, no difference was seen between HPV-positive and negative groups in sperm morphology, volume, and count [37]. Generally, HPV also has significant prevalence in men and is accompanied by a negative impact on quality of life.

#### 3.6.2. Vertical Transmission of HPV in Children

Evidence for vertical transmission of HPV is scarce and limited to very small cohorts. One study reported an overall HPV prevalence of 7.9% in children, with a higher rate observed after natural delivery (20.9%) compared with Cesarean section (4.4%), suggesting a potential role for the mode of delivery [129]. While some mother–child pairs showed shared genotypes (e.g., HPV16 or HPV18), discrepancies were also common, and one child tested positive despite having an HPV-negative mother [129]. Due to the extremely small sample size and reliance on a single study, these findings should be interpreted with caution and do not currently support vertical transmission as a major route of infection in the MENA region.

#### 3.6.3. HPV Infection in High-Risk Populations

The prevalence of oral and anal HRHPV in Female Sex Workers (FSW) was 14.9% and 60.8%, respectively, and in non-FSW women, these rates were 10.5% and 39.3%. Also, HIV positivity in these women was associated with an increased risk of oral and anal hrHPV. The condom usage rate in oral sex among HPV-positive women was zero [130].

### 3.7. The Role of HPV in Non-Cervical Malignancies

HPV infection detected in tumor tissue samples from malignancies other than cervical cancer has varying prevalence depending on the tissue type and studied population [5,6,7,8,9,10,11] (Table 4).

#### 3.7.1. Oral and Oropharyngeal Malignancies

HPV prevalence in Head and Neck Squamous Cell Carcinoma (HNSCC) is significantly higher in the oropharynx compared with the oral cavity. In 106 HNSCC samples, 24.5% were HPV positive, with 27.8% in the oropharynx and 17.6% in the oral cavity. The most common reported genotype was HPV16 (84.5%), followed by HPV18, HPV45, and HPV82. In these cancers, overexpression of the p16INK4A gene had a strong correlation with HPV DNA [5]. In a retrospective cohort study with 675 HNSCC samples, considering HPV-E6*I mRNA positivity, the percentage of HPV cases was 40.4% in the oropharynx, 1.8% in the oral cavity, and 2.2% in the larynx. In this study, HPV16 was the most common genotype in all cancers of this region [6]. In another study of 285 HNSCC patients, 3.5% were HPV positive, where HPV16 was also the most common [19].

A study focusing on the Middle East and North Africa region reported an overall HPV prevalence of 16% in all head and neck cancers. This prevalence was significantly higher in the salivary glands (20%) and tonsils (16%). The study also identified significant geographic variation, with the highest reported rates in Turkey (48%) and Palestine–Israel (31%) and the lowest rates in Yemen (2%) and Saudi Arabia (4%). Furthermore, analysis of trends over time suggested a possible decline in the prevalence of HPV-associated HNC in the region from 19% (1998–2010) to 12% (2015–2019) [131].

#### 3.7.2. Esophageal and Gastric Malignancies

In a study of 168 esophageal lesions, 27% were HPV positive in neoplastic cases and 41.2% in non-neoplastic cases. In the neoplastic group, HPV35, HPV39, and HPV45, and in the non-neoplastic group, HPV56, were the most common genotypes [7]. In 100 gastric cancer samples, 5% were HPV positive, with the most common genotypes being HPV16, HPV18, and HPV45. Also, a significant relationship was observed between tumor location and HPV infection, with the highest prevalence of 66.7% in the lower third of the esophagus and upper stomach [8].

The role of HPV in esophageal cancer has been investigated, especially in high-prevalence areas, such as Iran, which showed that 25.32% of esophageal squamous cell carcinoma cases were HPV positive, indicating an approximately 2-fold higher probability of HPV infection in cases compared with controls. In this context, HPV16 and HPV18 were the most common genotypes [132].

#### 3.7.3. Breast and Thyroid Malignancies

Evidence for the role of HPV in breast cancer in the Middle East and North Africa (MENA) region has been identified in several other studies. A case–control study from northern Iran found HPV DNA in 25.9% of breast tumors versus 2.4% of controls (OR = 14.2), with HPV16 and HPV18 being the predominant types [133]. In another study, HPV prevalence in patients with breast cancer was 22.2%, and HPV16 was the most common reported genotype at 87.5% [10]. In patients from Sudan with breast cancer, HPV prevalence was 8.7%, with reported genotypes including HPV16, HPV58, HPV18, and HPV11 [9]. A review of Iranian studies estimated the prevalence of HPV among breast cancer patients to be 23.6%, with an OR of 5.7 for developing breast cancer in HPV-positive women [134]. Furthermore, a study from Algeria reported that aggressive breast cancer subtypes, particularly inflammatory breast cancer (30% vs. 13%, *p* < 0.04) and triple-negative tumors (44% vs. 14%, *p* < 0.009), harbored significantly more viral DNA, with EBV1 and HPV16 being the most prevalent [135]. Additionally, HPV has been detected in other malignancies; for instance, 13.4% of papillary thyroid carcinoma samples were HPV-positive, significantly higher than in benign thyroid nodules [11].

## 4. Discussion

The present narrative review study examined the epidemiology, genotypic distribution, risk factors, awareness of HPV, vaccination status, and clinical outcomes of HPV infection in various populations across the Middle East and North Africa (MENA) region. Findings indicate that HPV prevalence in the general female populations ranges from 4.7% to 53.3% [32,33,34,35,36,37,38,39,40,41,42,43,44,45,46,47,48,49,50,51,52,53,54,55,56,57,58,59], rising to over 90% in high-risk populations [60,61]. This heterogeneity in reported figures likely reflects methodological differences, variation in the studied populations, socio-cultural factors, and differences in healthcare systems [32,33,34,35,36,37,38,39,40,41,42,43,44,45,46,47,48,49,50,51,52,53,54,55,56,57,59]. These findings are consistent with other review studies. In a global meta-analysis study, HPV prevalence was estimated at 10.4%, with Africa showing the highest prevalence at 22.1% and Asia the lowest at 8% [136]. A systematic review and meta-analysis focused on the MENA region similarly reported HPV prevalence of 81% among patients with cervical cancer [137].

Regarding genotypic distribution, HPV16 and HPV18 were the most common high-risk genotypes [35,36,39,40,46,50,53,59,63,64,66,67,68,69,70,71,72,73,74]; however, in some regions, other genotypes such as HPV33, HPV31, HPV35, HPV56, HPV39, HPV52, HPV51, and HPV45 predominated [36,43,61,63,75,76,77]. These deviations from global patterns cannot be attributed solely to methodological differences; they may reflect unique backgrounds, mating patterns, or historical migration patterns within the MENA population that influence viral evolution and transmission dynamics. Further molecular studies are needed to explore these host–pathogen interactions. Consistent with these findings, a systematic review of the Eastern Mediterranean region identified HPV16 as the most common genotype, followed by HPV18 [138].

Our review highlights a notable prevalence of co-infections with multiple HPV genotypes or concurrent sexually transmitted infections (STIs) in the MENA region, ranging from 0.5% to 53.8% [33,36,42,47,55,56,65,71,74,79,81,89]. This high rate of co-infection may facilitate viral persistence and accelerate disease progression. While the relationship between the number of infecting genotypes and lesion severity remains debated—some studies link multiple infections to higher-grade lesions [90], while others find no significant correlation [65]—our findings align with global meta-analyses indicating that co-infection with multiple high-risk types is associated with an increased risk of high-grade squamous intraepithelial lesions (HSIL) [139] and lesion recurrence [140]. Furthermore, the strong association between HPV and other STIs (e.g., *Chlamydia trachomatis*, HSV) underscores the shared transmission dynamics and potential synergistic effects on cervical carcinogenesis, warranting integrated screening approaches in clinical practice.

Consistent with global patterns, our analysis identifies younger age, smoking, alcohol consumption, early sexual debut, and multiple sexual partners as key risk factors for HPV acquisition in the MENA region [45,47,50,52,56,62,64,68,72,74,84,91,141,142]. Notably, the impact of behavioral factors such as tobacco use appears amplified in this population, with smokers showing significantly higher rates of persistent infection and severe cervical pathology compared with non-smokers [62,64,90]. This suggests that lifestyle interventions targeting smoking cessation could serve as a critical adjunct to HPV prevention strategies. Additionally, cultural shifts towards more liberal sexual behaviors among younger generations in the MENA region may contribute to the rising incidence observed in recent years, mirroring trends seen in Western countries before widespread vaccination.

A fundamental challenge in the MENA region is the gap between theoretical knowledge and practical vaccine uptake. While general public awareness of HPV is moderate to poor [23,24,25,27,51,77,94,95,96,97,98,99,101,109,110,143,144,145,146,147,148], even healthcare professionals—who possess relatively higher knowledge levels—demonstrate severely low vaccination rates [98,102,104,105,106,108,149,150,151]. This “knowledge-practice gap” indicates that barriers extend beyond education. Key deterrents include financial constraints, fear of side effects, and deep-seated socio-cultural stigmas linking vaccination to sexual promiscuity [26,27,28,29,113,114,115]. These barriers are compounded by the absence of national immunization programs in most of the studied countries. In contrast, nations like Turkmenistan and Uzbekistan have achieved coverage rates exceeding 90% through government-funded initiatives [111,116]. This stark contrast demonstrates that policy-level interventions, particularly financial support and programmatic inclusion, are more effective drivers of vaccine uptake than awareness campaigns alone.

In addition to cervical malignancies, our review confirms the presence of HPV DNA in tissues from head and neck, esophageal, gastric, breast, and thyroid cancers in the MENA region [5,6,7,8,9,10,11,19]. However, the strength of evidence varies considerably across these cancer types. For Head and Neck Squamous Cell Carcinoma (HNSCC), particularly in the oropharynx, the association is robust; prevalence rates range from 17.6% to 27.8%, with HPV16 being overwhelmingly dominant (84.5%) [5,6]. This pattern aligns with global trends in which HPV-driven HNSCC is increasingly recognized as a distinct clinical entity. In contrast, for esophageal and gastric cancers, reported prevalence estimates are lower and more heterogeneous (27% and 5%, respectively) [7,8]. While some systematic reviews suggest an association between HPV and esophageal cancer [152], the causal link remains controversial in the MENA context due to potential contamination from oral flora arising from the anatomical proximity of the oropharynx to the esophagus. Similarly, for breast and thyroid cancers, prevalence ranges widely (8.7–22.2% for breast [9,10]; 13.4% for thyroid [11]). These wide ranges likely reflect not only true geographic or biological heterogeneity but also significant methodological challenges, such as the lack of standardized detection protocols and the risk of false positives from blood contamination in tissue samples [Table 4]. Unlike cervical cancer, where HPV is a necessary cause, its role in extra-cervical malignancies may be opportunistic or co-factorial rather than primary. Therefore, future research must prioritize rigorous, multi-center studies using standardized PCR methods and validated biomarkers (e.g., p16 for HNSCC) to distinguish true viral oncogenesis from incidental colonization. Furthermore, investigating the interaction between HPV infection and local environmental carcinogens (e.g., dietary factors in gastric cancer) could generate novel hypotheses regarding synergistic carcinogenic effects in this region.

The present review has several strengths, including the collection of extensive data from Middle Eastern and North African countries and the simultaneous coverage of prevalence, genotype distribution, risk factors, public awareness, and vaccination status. In addition, distinguishing high-risk and low-risk genotypes and providing prevalence ranges based on the reviewed studies facilitates understanding of the dominant pattern in this region.

This study has several limitations that should be considered when interpreting the findings. First, there is significant geographical bias in the available literature; the majority of included studies originated from Iran and Saudi Arabia, while North African countries (e.g., Libya, Algeria, Morocco, Tunisia) and conflict-affected regions were severely under-represented. This limits the generalizability of our findings to the entire MENA region. Second, publication bias cannot be ruled out, as studies reporting significant associations or high prevalence are more likely to be published. Third, restricting the search to English-language articles may have introduced language bias, potentially excluding relevant local studies published in Arabic or Persian. Finally, the high heterogeneity in study designs, diagnostic methods (PCR vs. Hybrid Capture), and biological samples make direct comparison of results difficult. The wide ranges in prevalence estimates likely reflect both true epidemiological diversity and these methodological inconsistencies. Therefore, future research should focus on standardized, population-based surveillance systems to generate comparable data.

In summary, while the MENA region faces significant challenges regarding HPV prevalence, awareness, and vaccination coverage, these findings also highlight actionable pathways for improvement. Given the high burden of HPV-related diseases and the low vaccination coverage in the MENA region, immediate policy interventions are critical. First, governments must prioritize the inclusion of HPV vaccines in national immunization programs with full financial support to eliminate cost barriers, as evidenced by the high uptake rates in countries such as Turkmenistan and Uzbekistan [111]. Second, culturally tailored educational campaigns are essential to address deep-seated myths, religious concerns, and social stigma associated with sexual health, which currently hinder vaccine acceptance among parents and adolescents [30,113]. Third, healthcare systems should mandate HPV education for medical students and professionals to bridge the gap between knowledge and practice, ensuring that healthcare providers actively recommend the vaccine [102,153]. Finally, establishing robust national cancer registries and standardized screening protocols will enable accurate monitoring of genotype shifts and vaccine effectiveness, facilitating evidence-based adjustments to public health strategies over time.

## 5. Conclusions

This narrative review provides an overview of the epidemiological status of HPV in the Middle East and North Africa region, highlighting widespread prevalence and associated oncological outcomes. Evidence also supports a role for HPV in non-cervical malignancies and its presence in male populations. Nevertheless, given the substantial disease burden, the performance of health systems in the region remains suboptimal. Very low levels of awareness, cultural stigma, cost, and fear of vaccine side effects have contributed to persistently low vaccination coverage, even among healthcare professionals. In addition, the absence of national vaccination programs in most countries has limited opportunities for primary prevention and imposed a heavy economic burden on health systems through treatment costs. Therefore, to reduce HPV-related malignancies in the region, it is essential to establish national vaccination programs, implement culturally tailored education to improve public awareness, and expand screening coverage. Furthermore, given the heterogeneity of existing studies, more longitudinal research is needed to examine genotypic changes and the role of HPV in other malignancies.

## Figures and Tables

**Table 1:** Summary of Common Low-Risk and High-Risk Genotypes from Various Studies.

Classification	Genotype	General Female Population (Prevalence Among All Screened Women) (%) *	High-Risk & Clinical Populations (Prevalence Among Women with Abnormal Cytology, CIN, or ICC, or Among HPV-Positive Individuals) (%) **	Reference
High risk (HR-HPV)	HPV16	2.5–15.28	14.7–89.3	[35,44,46,47,49,50,52,54,55,61,62,63,67,68,70,71,73,74,77,78,79,80,81]
	HPV18	1.8–13.3	6.1–35.3	[35,40,43,44,46,47,50,54,55,61,66,67,68,70,71,73,74,76,77,78,80,81,82]
	HPV31	3–11	3.6–31.57	[43,44,63,66,67,68,71,73,74,76,78,82]
	HPV33	-	1.8–53.8	[50,60,63,68,71,74]
	HPV35	-	0–14.3	[43,44,63,67,68,71,74]
	HPV39	1.05–4.5	4.6–37.7	[38,43,60,61,63,66,67,68,74,76,83]
	HPV45	1.8–3.3	6.3–49.47	[35,43,61,63,66,67,68,74,80,81]
	HPV51	2.2–9.2	7.2–14.3	[43,44,50,68,71,73,74,76]
	HPV52	2.89–13	5.9–36.56	[33,36,43,44,50,61,63,66,67,68,74,76,79]
	HPV56	2–18	7.7–33.3	[36,43,50,68,71,74,76,77,84]
	HPV58	-	1.8–31.25	[43,51,52,67,68,74,85]
	HPV59	0.9–3.3	4.8–7.2	[67,68,74,76]
	HPV66	-	1.8–14	[43,44,68,76]
	HPV68	2.5–9.1	6.8–11.4	[43,61,67,68,69,74,76]
	HPV82	-	2	[50]
Low risk (LR-HPV)	HPV6	3.2–35.2	10.3–62.9	[33,35,36,38,41,44,50,51,64,66,69,74,76,78,79,84,86]
	HPV11	1–23	5.9–55.2	[33,35,36,38,59,61,64,69,74,76,79]
	HPV40	-	0.5–8.1	[38,39,74]
	HPV42	1.8–2.8	1–8.6	[44,74,79,87]
	HPV43	-	1–4.1	[74,87]
	HPV44	1.6–5.6	7.3–13.1	[36,61,74]
	HPV53	4.7	7.3–9.3	[36,44,59,65]
	HPV54	1.8–9	5–32.2	[39,69,74,76,79]
	HPV61	0.9–5.6	-	[36,74]
	HPV62	4.48–5.7	11.4	[33,66,74]
	HPV81	3–5.7	11.9	[44,56,66,74,88]

* “General female population” indicates prevalence among all screened women. ** “High-risk and clinical populations” indicates prevalence among women with cervical lesions or HPV-positive individuals. Note: Direct comparisons between columns are not valid due to different denominators. Ranges indicate study heterogeneity (geography, sample size, assays).

**Table 4:** Prevalence and Clinical Implications of HPV in Non-Cervical Malignancies in the MENA Region.

Cancer Type	Approximate HPV Prevalence in MENA Studies	Key Evidence & Predominant Genotypes	Diagnostic Challenges & Interpretation Notes
Head and Neck Squamous Cell Carcinoma (HNSCC) (Oropharynx vs. Oral Cavity)	• Oropharynx: ~27.8% • Oral Cavity: ~17.6% (Overall HNSCC: ~24.5%)	• HPV16 is overwhelmingly predominant (84.5%). • Strong correlation between HPV DNA and p16INK4a overexpression. • Higher prevalence in oropharynx compared with oral cavity/larynx [5,6,19].	• p16 as a surrogate marker: While p16 is used as a proxy for HPV-driven cancers, false positives can occur. • Site-specificity: Distinguishing primary oropharyngeal cancer from other head/neck sites is crucial as HPV status drastically changes prognosis and treatment.
Esophageal Cancer	• Neoplastic lesions: ~27% (Note: Some studies report higher rates in non-neoplastic tissue, suggesting colonization vs. causation)	• High-risk genotypes: HPV35, HPV39, HPV45. • Association with tumor location (highest in lower esophagus/upper stomach). • Co-infection with EBV observed in some studies [7,8].	• Contamination risk: Esophageal samples are prone to contamination from oral flora/oropharyngeal HPV, which may lead to overestimation of causal role. • Causality vs. Passenger: Difficulty in distinguishing whether HPV is driving carcinogenesis or merely present due to anatomical proximity to the oropharynx.
Gastric Cancer	• Low prevalence (~5%) (Significant relationship with tumor location in lower third of esophagus/upper stomach)	• Common genotypes: HPV16, HPV18, HPV45. • Evidence is limited compared with cervical/HNSCC [8].	• Low viral load: Detection rates are low, making statistical power difficult. • Methodological variability: Differences in PCR primers and sample types (tissue vs. blood) affect comparability across studies.
Breast Cancer	• Range: 8.7–22.2% (Highly variable across studies)	• HPV16 is the most frequently reported genotype (up to 87.5% in some Egyptian cohorts). • Association with ER/PR status noted in some studies [9,10].	• Blood Contamination: Breast tissue samples often contain residual blood, which contains circulating HPV DNA, leading to potential false positives if not rigorously controlled. • Inconsistency: Wide range in prevalence suggests lack of standardized detection methods or true biological heterogeneity.
Thyroid Cancer (Papillary Carcinoma)	• ~13.4% in Papillary Thyroid Carcinoma (Significantly higher than in benign nodules)	• Presence of HPV DNA linked to malignant transformation. • Potential association with tumor staging and pathologic features [11].	• Benign vs. Malignant distinction: Need to ensure that HPV detection is specific to malignant cells and not just incidental presence in thyroid tissue. • Small sample sizes: Many studies on thyroid HPV are based on small cohorts, limiting generalizability

## Data Availability

No new data were created or analyzed in this study. Data sharing is not applicable to this article.

## Supplementary Materials

The following supporting information can be downloaded at: https://www.mdpi.com/article/10.3390/diseases14080302/s1, Table S1: Summary of studies included in the narrative review.
