# Supplementary material for: Epidemiology of Human Papillomavirus in the Middle East and North Africa: A Review of Genotypic Prevalence, Vaccination Challenges, and Non-Cervical Clinical Outcomes
Source: Diseases. 2026 Aug 19;14(8):302. doi: 10.3390/diseases14080302 (PMC13512765; doi:10.3390/diseases14080302)
Supplement: Supplementary file 1 [file diseases-14-00302-s001.zip › diseases-4465737-supplementary.pdf]

Table S1. Summary of studies included in the narrative review.

| First author (year) | Type of study | Sample size | Country | Key finding | Outcomes |
|---|---|---|---|---|---|
| Alhamlan et al. (2021) [1] | Review | N/A | Global | HPV causes ~4.5% of all cancers; prophylactic vaccines reduce infection burden. | HPV vaccination and screening are essential; non-cervical HPV cancers are increasing in developed countries. |
| Shalchimanesh et al. (2022) [2] | Case series | 219 | Iran | HR-HPV (67.12%) more common than LR-HPV; HPV-16 (17.4%), HPV-68 (11.4%), HPV-6 (31.1%) most frequent | Remarkable growth of HPV-68 reported; highest prevalence in <30 age group; co-infection with multiple HPV types in 22.4% |
| Aliabadi et al. (2025) [3] | Cross-sectional | 354 | Iran | Human Papillomavirus prevalence 16.9% in females (12.5% high-risk) and 13% in males (40% high-risk); reduced assisted reproductive technology use in positive couples. | Human Papillomavirus-positive females had lower age at marriage; positive males had higher self-employment and partner change but lower smoking; no significant differences in semen parameters. |
| Moradi et al. (2025) [4] | Cross-sectional | 872 | Iran | Overall prevalence 38.9%; highest in males (64.3%) and wart lesions; most common genotype Human Papillomavirus-6 (62.9%), most common high-risk Human Papillomavirus-50s (26.5%). | Prevalence in vaginal swabs 37.4%, female genital warts 61.5%, males 64.3%; single infection 63.4%, double 30%, multiple 6.3%; Human Papillomavirus-6 most common in single infections (35.9%). |
| Mojarrad et al. (2025) [5] | Cross-sectional | 447 | Iran | Overall prevalence 43.6%; most common genotypes Human Papillomavirus-6 (41.0%), -16 (15.4%), -56 (10.8%), -90 (10.8%); peak in under 30 and 31–40 age groups. | Prevalence females 42.5%, males 75.0%; single infection 46.2%, multiple 53.8%; low-risk genotypes more common than high-risk; Human Papillomavirus-6 most frequent in mixed infections. |
| Küçükyurt et al. (2025) [6] | Retrospective observational | 842 | Turkey | Age affects cervical intraepithelial neoplasia detection; women aged 18-25 have higher likelihood of Cervical Intraepithelial Neoplasia 3 diagnosis than those 26-29. | Abnormal cytology 11.6%; Human Papillomavirus positivity 8.9% (53.3% of tested); colposcopy confirmed Cervical Intraepithelial Neoplasia+ in 38/53; Cervical Intraepithelial Neoplasia 3 less frequent in >26 group on both colposcopy and conization. |
| Aygar et al. (2025) [7] | Retrospective cross-sectional | 7009 | Turkey | Prevalence 11.3%; other high-risk types most common (66.8%); Human Papillomavirus-16 (15.3%), Human Papillomavirus-18 (5.8%); peak in 18-19 years (33.3%). | Positivity declines with age; monthly positivity decreased over time; 86.4% viral clearance post-treatment; 12.7% in pregnant women. |
| El-Daly et al. (2025) [8] | Cross-sectional | 106 | Saudi Arabia | Prevalence 15.1%; Human Papillomavirus-16 most common (43.75% of positives), then -58 (31.25%), -6 (25%); peak in 35-44 years. | High-risk types 43.75%; non-Saudis higher prevalence (40% vs. 12.5%); irregular menstruation significantly associated; >60% unaware of Human Papillomavirus-cervical cancer link. |

| | | | | | |
|---|---|---|---|---|---|
| Tanik et al. (2025) [9] | Descriptive cross-sectional | 3510 | Turkey | Prevalence 10%, peak 18-24, other high-risk common, 33-fold Low-Grade Squamous Intraepithelial Lesion/High-Grade Squamous Intraepithelial Lesion risk. | Single 86%, multiple 14%; abnormal 6% (Atypical Squamous Cells of Undetermined Significance 5%, Low-Grade Squamous Intraepithelial Lesion 0.9%, High-Grade Squamous Intraepithelial Lesion 0.5%); Human Papillomavirus in Low-Grade Squamous Intraepithelial Lesion 77%, High-Grade Squamous Intraepithelial Lesion 75%. |
| Sadeghi et al. (2024) [10] | Cross-sectional | 258 | Iran | Human Papillomavirus type 16 increases cancer risk; Epstein-Barr virus lowers risk. | Human Papillomavirus type 16 positivity and load rise with severity; Human Papillomavirus type 18 load not significant; Epstein-Barr virus prevalence and load higher in controls. |
| Letafati et al. (2024) [11] | Cross-sectional | 428 | Iran | Overall prevalence 30.1% (females 30.1%, males 30.4%); most common genotypes Human Papillomavirus-6 (10.3%), -16 (8.7%), -51 (7.7%), -39 (6.7%); positive cases had significantly lower mean age. | 49.4% had at least one high-risk genotype, 29.2% low-risk, 21.3% potential high-risk; males exclusively low-risk; single infection 69%, multiple 31%; Human Papillomavirus-18 prevalence 0.5%. |
| Letafati et al. (2024) [12] | Cross-sectional | 2109 | Iran | Prevalence 17%; most common Human Papillomavirus-16 (11.7%), -54 (10.3%), -56 (8.4%); peak in under 30 (35%). | 46% low-risk, 45% high-risk; under 30 predominantly high-risk; 77% single infection, 23% multiple. |
| Gholamzad et al. (2024) [13] | Cross-sectional | 936 | Iran | Prevalence 34.6%; common 16, 6, 11, 18; vaccine reduces covered types but uncovered may rise. | Males 27.5%, females 37.3% positive; males predominantly HR, females predominantly LR; vaccinated: all 49 genotypes were HR (no 16/18). |
| Etemad et al. (2024) [14] | Cross-sectional (retrospective) | 328 | Iran | Prevalence 37.5%; most common Human Papillomavirus-6 and -16; inflammation and endocervical cells increase Human Papillomavirus odds. | 48.7% single genotype; Human Papillomavirus-positive women younger (31.5 vs. 39.1 years); adjusted odds ratios: endocervical cells 25.30, inflammation 11-19. |
| Haddadi et al. (2024) [15] | Cross-sectional | 950 | Iran | Overall prevalence 26%; 69% of positives were high-risk; most common genotypes 6, 16, 52, 53, 51, 58, 56; Human Papillomavirus-16 (17%) and -52 (13%) highest high-risk. | Females 25% positive, males 76% positive; highest number of positives in 31-40 years; under 20 highest percentage (40%); Human Papillomavirus-33 not detected; Human Papillomavirus-35 and -59 lowest (3% each). |
| Dura et al. (2024) [16] | Retrospective cohort | 10152 | Turkey | Prevalence 10.9%; Human Papillomavirus-16 most common (52% in colposcopy group); primary Human Papillomavirus test more sensitive than smear. | Premenopausal 9.8%, postmenopausal 12.4%; Human Papillomavirus test sensitivity 90-92%, specificity 58%; co-test sensitivity 97-100%, specificity 28%. |

| | | | | | |
|---|---|---|---|---|---|
| Heidari Pebdeni et al. (2023) [17] | Case-control | 190 (95 infertile, 95 fertile) | Iran | Human Papillomavirus significantly increases infertility risk (adjusted odds ratio 7.02). | Human Papillomavirus prevalence 14.7% in infertile vs. 3.2% in fertile; bacterial agents not significantly different between groups. |
| Bakhshani et al. (2023) [18] | Cross-sectional (retrospective) | 1214 | Iran | Prevalence 31.8%; most common high-risk 31, 16, 51; low-risk 6, 53, 42; multiple infections (12.4%) > single (10.4%). | Annual prevalence increased 14-fold; Human Papillomavirus-6 was 24% of all; 37 genotypes detected. |
| Taskin et al. (2022) [19] | Retrospective cross-sectional | 5406 | Turkey | Prevalence 9.17%; other high-risk types most common (78.83%); Human Papillomavirus-16 (28.62%), -18 (9.67%). | Mean age 39.56 years; peak in 40-49 years; no significant age-group differences. |
| Tekkesin et al. (2022) [20] | Retrospective cross-sectional | 5975 | Turkey | Prevalence 46.4%; Human Papillomavirus-16 most common (19.2%), -18 (10.8%), -51 (9.7%), -56 (7.7%), -52 (7.5%). | Peak under 31 years (45%); single infections 64%, multiple 36%; Human Papillomavirus-52 highest mean viral load (5.38 log); high-risk types 118.8% frequency. |
| Saeed et al. (2025) [21] | Retrospective comparative | 320 | Bahrain | Polymerase Chain Reaction more sensitive (95.5%), less specific (67.4%) than Pap; combining improves detection. | Human Papillomavirus prevalence 17.5%; positive predictive value 94.6%, negative predictive value 96.9%; abnormal cytology 42.5% (higher in Human Papillomavirus+). |
| Sait et al. (2024) [22] | Cross-sectional + cohort | 5360 | Saudi Arabia | Prevalence 4.7%; 84% clearance at 1 year; 0.2% new at 5 years; parity inversely linked. | Cumulative 5%; incidence 47/100,000; Cervical Intraepithelial Neoplasia 2+ in 0.3%; older/divorced lower clearance. |
| Tavakoli Dastjerd et al. (2025) [23] | Cross-sectional | 80 | Iran | Human Papillomavirus prevalence 76% in Human Immunodeficiency Virus+; Human Papillomavirus-39 & -52 most common; 71% multiple high-risk. | High-risk 67%, low-risk 31%; abnormal cytology 7.5% (all high-risk); Human Papillomavirus-44 most low-risk (13%); no link to CD4, antiretroviral therapy, or risk factors. |
| Banjer et al. (2025) [24] | Retrospective national laboratory-based | 9096 | Saudi Arabia | Positivity 13.84%; non-16/18 dominant (67%); peak 30-39 years; Jeddah/Riyadh highest. | Human Papillomavirus-16 25%, Human Papillomavirus-18 7.5%; precancer 13%, cancer 0.6%; 19% Human Papillomavirus+ had normal cytology; positivity rose 5.6%→13.3% over 8 years. |
| El-Daly et al. (2025b) [25] | Cross-sectional | 260 | Saudi Arabia | Prevalence 6.9%; Human Papillomavirus-16 & -6 each 33%; peak 31-40 years. | High-risk 56%, low-risk 44%; Saudis 6%, non-Saudis 9%; 89% Human Papillomavirus+ had normal cytology. |
| El-Wakil et al. (2024) [26] | Cross-sectional | 74 | Egypt | Prevalence 37.8%; Human Papillomavirus-16 dominant (89%); high nitric oxide linked to high-risk Human Papillomavirus & high-grade atypia. | Atypia 17% (higher in high-risk+); nitric oxide higher in high-risk+ (37 vs 2.3); cut-off >23.6 predicts high-risk (area under the curve 0.97). |
| Abdoudaim et al. (2025) [27] | Cross-sectional | 50 | Mauritania | Tissue Human Papillomavirus viral load higher in invasive cervical cancer than Cervical Intraepithelial Neoplasia 2/3 and distinguishes them. | Any Human Papillomavirus and high-risk Human Papillomavirus loads significantly higher in invasive cervical cancer; optimal cutoffs 4.38 (any Human Papillomavirus) |

| | | | | | |
|---|---|---|---|---|---|
| | | | | | and 4.85 (high-risk Human Papillomavirus) copies/10,000 cells. |
| Ashry et al. (2024) [28] | Cross-sectional | 519 (251 Women Living With Human Immunodeficiency Virus, 268 Human Immunodeficiency Virus-negative) | Egypt | Prevalence 13.5% (Human Immunodeficiency Virus-positive 24.4%, Human Immunodeficiency Virus-negative 3.4%); other high-risk most common (71%). | Predictors: age (odds ratio 0.96), marriage age (odds ratio 0.91), multiple marriages (odds ratio 2.07), drug addiction (odds ratio 2.01). |
| Nadri et al. (2025) [29] | Cross-sectional | 31 | Iran | HPV-6 (44.8%) and HPV-11 (55.2%) were the predominant genotypes in RRP patients | HPV infection in RRP showed significant correlation with suburban residence, low income, and welfare status, with pediatric patients predominantly affected |
| Sadeghi et al. (2026) [30] | Cross-sectional | 220 | Iran | CCNE1 and CDK2 gene expression was significantly upregulated in HPV-positive patients, especially HPV-16 and HPV-18 | High-risk HPV genotypes are associated with increased transcription of cell cycle genes, and cervical microbiome shifts show decreased Lactobacillus with increased Prevotella_7 and Megasphaera |
| Aslanimehr et al. (2025) [31] | Retrospective | 103 | Iran | HPV-16 (66.31%) was the most common genotype, followed by HPV-45 (49.47%) and HPV-33 (41.05%) | HPV-16 was significantly associated with severe lesions (92% in ICC cases), and multiple infections (82.11%) were more common than single infections |
| Soleimani-Jelodar et al. (2024) [32] | Cross-sectional | 148 | Iran | Total HPV prevalence was 92.5%, with HPV-16 (49.3%) and HPV-18 (14.8%) being the most common | HPV-16 and HPV-18 were dominant in all histopathological stages, detected in 80% of cervical cancer samples, indicating current vaccines would be effective in Iran |
| Moeinzadeh et al. (2020) [33] | Cross-sectional | 550 | Iran | HPV-53 and HPV-16 were the most prevalent genotypes, with HR-HPV infection rate of 78% | HPV-53 was the most frequent genotype alongside HPV-16, and six new HPV genotypes (61, 62, 70, 81, 83, 89) were identified in Iranian population |
| Letafati et al. (2024) [34] | Cross-sectional | 90 | Iran | HPV prevalence was 67.7%, with HPV-11 (23%), HPV-6 (21%), HPV-56 (18%), and HPV-39 (16%) most common | Highest HPV prevalence (75%) was in individuals under 31 years; HPV-56 and HPV-39 were the most common high-risk genotypes, not covered by current vaccines |
| Yerim et al. (2025) [35] | Comparative | 979 | Morocco | HPV prevalence was 4.0% by PCR and 5.0% by Xpert, with 97.5% concordance between methods | Xpert HPV assay showed excellent analytical performance (sensitivity 82.1%, specificity 98.2%), making it reliable for primary cervical cancer screening |
| Çubuk et al. (2025) [36] | Comparative | 1200 | Turkey | HPV DNA was detected in 11.9% by nucleic acid extraction and 11.4% by PharmaDirect method | PharmaDirect showed 88.1% sensitivity compared to nucleic acid extraction, with "great agreement" (Kappa=0.868), offering an alternative for population-based screening |

| | | | | | |
|---|---|---|---|---|---|
| Ben Taher et al. (2025) [37] | Validation study | 134 | Tunisia | Xpert HPV showed 99.16% sensitivity and 100% specificity for HR-HPV detection on FFPE tissues | HPV-16 (71%) was most common, followed by HPV-31 (8%) and HPV-18 (5%); Xpert HPV showed almost perfect agreement (κ=0.892) with multiplex PCR |
| Guzelbag et al. (2026) [38] | Retrospective | 83,148 | Turkey | Overall ECA rate was 7.93%; HPV positivity was 9.1% (mRNA) and 9.9% (DNA); HPV-16 was most prevalent | Significant decrease in ECA rates (r=-0.591, p=0.043) and high-grade abnormalities (r=-0.911, p<0.001) over 12 years; HPV-56 and HPV-51 were more prevalent than HPV-18 |
| Tahir et al. (2025) [39] | Retrospective | 2,193 | Iraq | Overall HPV prevalence was 12.7%, with HPV-18 (13.3%) most common, followed by HPV-11 (11.4%) and HPV-16 (6.9%) | Single infections (83.5%) were more common than multiple infections (16.5%); HR-HPV (6.9%) was more frequent than LR-HPV (4.4%) |
| Salhi et al. (2025) [40] | Retrospective | 48 | Palestine | HPV was detected in 82.5% of samples, with high-risk types in 77.5%; HPV-16 (32.5%) and HPV-18 (10%) most prevalent | Highest HPV positivity was in women aged 50-59 years; geographic variation in HPV genotype distribution was observed across Palestinian regions |
| Sadeghi et al. (2025) [41] | Cross-sectional | 710 | Iran | HPV-16 (43.7%) was most prevalent; CIN was found in 52.4% of HPV-positive women | HPV-16 (OR=2.88) and smoking (OR=1.53) were significant predictors of severe CIN; older age and later sexual debut correlated with better outcomes |
| Nikkhoo et al. (2025) [42] | Cross-sectional | 2,650 | Iran | Overall HPV prevalence was 25.1%; HPV-6 (30.1%), HPV-16 (14.4%), and HPV-54 (9.0%) most common | HR-HPV was found in 45.6% of cases; highest prevalence in 30-34 age group; superinfection in 36.2% of participants |
| Kılıç et al. (2025) [43] | Retrospective | 643 | Turkey | HPV positivity was 55.5%; HPV-16 (24.6%) most common; SCC was 3 times more frequent in HPV-16/18-positive cases | Full cytology-histology concordance was 51.6%; diagnostic discordance increased with age and HPV negativity; SCC was most common in 30-65 age group |
| Alci et al. (2025) [44] | Retrospective | 882 | Turkey | HPV-16 only in 59.4%; HPV-16 with other genotypes in 40.6% of patients | No significant difference in histopathological outcomes between HPV-16 only and co-infection groups; smoking increased co-infection risk 1.27-fold |
| Gunes et al. (2023) [45] | Prospective | 9,808 | Turkey | Colposcopy was performed in 90.1% of non-required patients; ≥CIN-3 detected in 15.9% of required and 1.5% of non-required groups | High rates of unnecessary colposcopies (90.1%) and multiple biopsies (69.8%); guideline showed 94.1% sensitivity for ≥CIN-3 detection |
| Shabanpour et al. (2025) [46] | Cross-sectional | 134 | Iran | HPV-33 sublineage A2 (85.8%) and HPV-35 sublineage A1 (84.8%) were dominant | Four mutations detected in HPV-33 E6; seven mutations in HPV-35 E6; no significant association between sublineages and histology/cytology status |
| Samaria et al. (2025) [47] | Cross-sectional | 441 | Indonesia | Significant differences in self-efficacy and knowledge among decision-making groups | SDM had highest median self-efficacy (15) and knowledge (34); HDM had lowest median self-efficacy (11) and knowledge (24); P<0.001 for both |

| | | | | | |
|---|---|---|---|---|---|
| Falahi et al. (2025) [48] | Cross-sectional | 108 | Iran | HPV DNA detected in 10.18% of blood donors | Genotype 16 in 3 cases; genotype 6 in 2 cases; genotypes 11,31,35,51,53,59 in 1 case each; positive cases included 4 men and 7 women aged 25-37 years |
| Salhi et al. (2025) [40] | Retrospective | 40 | Palestine | HPV detected in 82.5% of cervical cancer samples | HPV16 (32.5%) and HPV18 (10%) most prevalent; high-risk HPV types 77.5%; highest HPV positivity in women aged 50-59 years |
| Abi Aad et al. (2024) [49] | Cross-sectional | 38 | Lebanon | HPV detected in 89% of men with HIV | Multiple strains in 91%; HPV16 most common (56%); 18% had bacterial STIs; 75% unaware of anal dysplasia risk; only 29% vaccinated |
| Khezeli et al. (2025) [50] | Cross-sectional | 120 | Iran | HPV16 lineage D was predominant (79.2%) | Lineages A (20%) and C (0.8%) also found; D1/4 sublineages dominant (79.2%); no significant association between histopathological stages and lineages |
| Shafaei et al. (2023) [51] | Cross-sectional | 190 | Iran | 34 different HPV genotypes detected; HPV6 most common | HPV6 (26.6%), HPV16 (19.5%), HPV52 (12.5%); high-risk HPV prevalence 64.84%; multiple infections 46.87%; smoking increased HPV risk 5.43 times |
| Mortazavi et al. (2021) [52] | Cross-sectional | 280 | Iran | No significant association between HPV and G. vaginalis/S. agalactiae | U. urealyticum 38.13% in HPV+ vs 29.62% in HPV-; N. gonorrhoeae 5.93% in HPV+ vs 3.08% in HPV-; C. trachomatis only in HPV+ (0.84%) |
| Mendes de Oliveira et al. (2025) [53] | Prospective cohort | 9,014 | Mozambique | HR-HPV prevalence 31.3%; higher among WLWH (39.7%) | HPV16 (16.9%), HPV18/45 (24.6%), other HR-HPV (77.1%); multiple HR-HPV groups in 17.8%; HPV16 most frequent in cervical cancer (56.7%) |
| Azadehrah et al. (2024) [54] | Cross-sectional | 500 | Iran | Significant correlation between TV infection and HPV infection | TV prevalence 29.2%; HPV prevalence 19.4% (HR-HPV 7.6%, LR-HPV 11.8%); significant association between TV and abnormal Pap smear ($P<0.001$) |
| Sadati et al. (2025) [55] | Cross-sectional | 291 | Iran | Low-risk HPV genotypes most common in genital warts (91.35%) | HPV6/11 in 91.4%; HPV16 in 6.92%; HPV18 in 1.73%; significant association between sex and genotype distribution ($P=0.003$); high-risk genotypes more prevalent in males (18.6%) than females (6.8%) |
| Zaky et al. (2025) [56] | Cross-sectional | 100 | Egypt | Low FSFI scores indicating sexual dysfunction in women with GWs | Total FSFI score 17.64±6.15; lowest scores in orgasm (1.91±1.07); significant impact on QOL in 49% of participants; disease duration significantly correlated with FSFI and DLQI |

| | | | | | |
|---|---|---|---|---|---|
| Farhadi et al. (2023) [57] | Cross-sectional | 648 | Iran | HPV DNA detected in 62.19% of screened subjects | HPV16 (17.6%), HPV56 (12.6%), HPV66 (12%); HPV6 most common LR-HPV (45.6%); multiple infections in 57.3%; HPV16 D1 sublineage (45.83%) and A4 sublineage (27.1%) most common |
| Alwadai et al. (2025) [58] | Cross-sectional | 261 | Saudi Arabia | 49.4% of nursing students had poor HPV knowledge | Moderate knowledge 47.1%; high knowledge 3.4%; mean attitude score 2.79±0.87; significant differences in knowledge based on living arrangement (P=0.025); no differences by academic year or parental education |
| Alshehri et al. (2023) [59] | Cross-sectional | 773 | Saudi Arabia | 46.1% of parents had poor HPV knowledge | Good knowledge 38.5%; 44.5% intended to vaccinate; 43.6% unsure; 11.9% no intention; positive correlation between knowledge and intention (r=0.209; P<0.01); internet main information source (46.4%) |
| Solak et al. (2025) [60] | Cross-sectional | 105 | Turkey | HPV awareness 70.5%; cervical cancer awareness 38.1% | HPV vaccination rate 10.5% (women 24% vs men 6.2%; P=0.021); barriers to vaccination: cost (60%), lack of information (45.7%), perceived harmfulness (45.7%); women more willing to vaccinate children (84% vs 58.8%; P=0.039) |
| Elmanzlawey et al. (2025) [61] | Cross-sectional | 1,431 | Egypt | HPV awareness among medical students was 91% | Good knowledge 30.3%; 78% believed vaccine causes serious adverse effects; 70-76% thought vaccine only for sexually active; males more willing to vaccinate (44% vs 36%; P=0.02); older age, urban residence, and prior HPV education predicted awareness |
| Alghamdi et al. (2025) [62] | Cross-sectional | 400 | Saudi Arabia | HPV awareness 49.3%; vaccine awareness 44% | Good knowledge 30.3%; vaccination rate 3.8%; willingness to accept vaccine 33.5%; older age (OR:1.42), maternal education (OR:4.65), and COVID-19 vaccination (OR:3.12) predicted HPV awareness; males less willing to vaccinate (OR:0.38; P=0.003) |
| Erbıyık et al. (2021) [63] | Cross-sectional | 700 | Turkey | 44.1% knew cervical cancer cause; 42.8% did not | 55.9% of those with regular doctor control knew HPV vaccine prevents warts and cervical cancer vs 38.5% without; 63.4% men and 75.4% women did not know vaccine effectiveness against penile/anal/laryngeal cancer |

| | | | | | |
|---|---|---|---|---|---|
| Cheema et al. (2024) [64] | Cross-sectional | 398 | Qatar | 22.4% had poor knowledge, 6.3% vaccinated, 71% of unvaccinated willing if recommended by healthcare provider. | Targeted campaigns and integration into childhood vaccination programs are needed. |
| Al-Antary et al. (2025) [65] | Cross-sectional | 295 | USA (Arab Americans) | Awareness of HPV-associated cancers ranged from 37.9% to 50.5%; males and those with lower education had lower awareness. | Culturally tailored interventions are necessary to improve awareness. |
| Tehranian et al. (2024) [66] | Cohort | 250 | Iran | HPV infection was associated with sexual dysfunction and lower quality of life; FSFI and SQOL scores improved over 12 months. | HPV infection can impair sexual function and quality of life. |
| Doğan et al. (2024) [67] | Descriptive cross-sectional | 322 | Turkey | Male HCWs had good HPV knowledge but poor vaccine knowledge; vaccination rate was 6.3%; willingness correlated with knowledge. | Sociodemographic, cultural, and behavioral factors influence HPV vaccination. |
| Alsulami et al. (2024) [68] | Cross-sectional | 1149 | Saudi Arabia | Low HPV knowledge (mean 4.19/14); unmarried and graduate-educated had higher knowledge; knowledge positively associated with perceived benefits and negatively with barriers. | Enhanced knowledge boosts positive attitudes and reduces perceived barriers to vaccination. |
| Alherz et al. (2024) [69] | Cross-sectional | 390 | Saudi Arabia | 78.97% heard of cervical cancer, 50% HPV, 41.28% vaccine; overall poor knowledge; 60% willing to vaccinate daughters. | Awareness of HPV and vaccine is associated with parents' education level. |
| Abu-Elenin et al. (2025) [70] | Cross-sectional multicentric | 1300 | Egypt | 25% had good knowledge, 49.2% positive attitudes; most never vaccinated or screened; barriers included lack of awareness and no symptoms. | Older age and urban residence predicted negative attitudes; accessibility to screening needs improvement. |
| Shakurnia et al. (2025) [71] | Cross-sectional | 440 | Iran | Mean HPV knowledge score was 13.41 out of 20; 63.4% had adequate knowledge | Physician profession (OR=3.85, p=0.001), female gender (OR=1.61, p=0.001), being married, and age >30 years (OR=0.916, p=0.003) were significant predictors of adequate knowledge |
| Yörük et al. (2016) [72] | Cross-sectional | 725 | Turkey | Average total knowledge score was 14.15±6.7; only 0.9% received HPV vaccine | HPV vaccine knowledge score higher among faculty of medicine students; students with knowledge score above average (OR:1.5) and with family cancer history (OR:1.6) more likely to consider vaccination |
| Azzi et al. (2025) [73] | Cross-sectional | 858 | Saudi Arabia | HPV awareness increased from 38.8% in 2022 to 71.1% in 2024; HPV vaccine awareness from 28.2% to 58.7% | 67.6% willing to receive free HPV vaccination; only 25.8% had undergone Pap smear; knowledge about vaccine for males (AOR=8.48) and children (AOR=8.70) predicted awareness |

| | | | | | |
|---|---|---|---|---|---|
| Mahmoud et al. (2024) [74] | Cross-sectional | 831 | GCC countries | UAE had highest vaccination rate (18.9%); KSA 4.6%; Qatar 5.8% | Health insurance associated with higher vaccination (11% vs 5.4%, p=0.006); lack of knowledge (53.6%) and absence of medical recommendations (13.2%) were main barriers |
| Gari et al. (2023) [75] | Cross-sectional | 422 | Saudi Arabia | Mean knowledge score 7.3±2.31 out of 14; 36.3% heard about HPV vaccine; only 4.4% vaccinated | 39.6% declined HPV vaccine; reasons included not hearing about vaccine (35.3%), fear of side effects (30.5%), apprehension about injection (16.2%); students in health specialties had highest knowledge (p<0.001) |
| Varer Akpinar et al. (2023) [76] | Cross-sectional | 824 | Turkey | Mean HPV knowledge score was 6.74±7.13 out of 29; 43.6% never heard of HPV; 2.7% vaccinated | Being female, studying health sciences, being senior student, having had sex, and willingness to be vaccinated independently associated with higher knowledge (p<0.05); only 15.7% willing to be vaccinated |
| Tobaiqy et al. (2023) [77] | Cross-sectional | 500 | Saudi Arabia | 11% had heard HPV associated with cervical cancer; 96.8% never heard of HPV vaccine; 94% unwilling to vaccinate daughters | Primary reason for refusal was lack of information (85.2%); 83.8% believed insufficient information on vaccine safety and efficacy; married parents more likely to accept vaccination (p=0.02) |
| Abdelhadi et al. (2025) [78] | Cross-sectional | 304 | Jordan | Positive attitudes (OR=3.89) and active clinical practice (OR=5.02) were strong predictors of vaccine recommendation; higher knowledge paradoxically associated with reduced likelihood (OR=0.44) | 64.5% recommended HPV vaccine; significant KAP variation across professional groups; knowledge had weak negative correlation with attitudes (r=-0.25) and practices (r=-0.22) |
| Alghanmi et al. (2026) [79] | Cross-sectional | 261 | Saudi Arabia | Good knowledge score associated with higher vaccine acceptance (84.2% vs 67.3%, p=0.04); acceptance increased with education (46.7% to 89.3%) and income (45.2% to 77.7%) | Fear of side effects was main reason for refusal (48.1%); recommendations from MOH/doctor encouraged 92.2% of acceptors; 59.5% of refusers said more information on benefits/safety would encourage acceptance |
| Taumberger et al. (2025) [80] | Study protocol | 1,000 (planned) | Ghana | Protocol to evaluate hrHPV self-sampling screening program clinical efficacy | Primary outcome: proportion of women successfully screened; secondary outcomes include screening uptake, disease detection rate, HPV genotype prevalence, treatment acceptance, and disease recurrence |
| Larijani et al. (2025) [81] | Cross-sectional | 201 children + 74 mothers | Iran | Oral HPV prevalence 7.9% in children; HPV16 (50%) and HPV18 (37.5%) most common; maternal HPV positivity significantly increased child infection risk (OR=165, p<0.001) | Multiple infections in 43.75% of HPV-positive children; lack of HPV type concordance between mothers and children suggests horizontal transmission |
| Kayar et al. (2025) [82] | Matched case-control | 624 (312 smokers, 312 non-smokers) | Turkey | Smokers had higher abnormal cytology rates; >20 pack-years: 2.19x higher | Smoking significantly exacerbates HPV persistence and progression; heavy smoking |

| | | | | | |
|---|---|---|---|---|---|
| | | | | colposcopic abnormalities (p=0.027); >13 pack-years: 2.08x higher ECC abnormalities (p=0.008) | independently increases cervical neoplastic changes |
| Darvishali et al. (2025) [83] | Retrospective cross-sectional | 120 | Iran | HPV16 single infection most prevalent (66.66%); age was only independent predictor of overall survival (HR=1.035, p=0.020); HPV genotype not statistically significant in multivariable analysis | HPV genotype-specific trends suggest possible differences in survival, particularly for HPV18-containing infections; findings exploratory and hypothesis-generating |
| Alghalyini et al. (2024) [84] | Cross-sectional | 442 | Saudi Arabia | 54.1% aware of HPV, 66.5% heard of cervical cancer, only 17% correctly identified HPV16/18 as high-risk, only 10% vaccinated; females had significantly higher knowledge than males (p=0.018) | Low knowledge and vaccine uptake; main barrier: lack of education and awareness (80.1%); need for targeted educational programs |
| Faqeeh et al. (2024) [85] | Cross-sectional | 600 | Saudi Arabia | 49.7% familiar with HPV, 85.8% believed HPV causes serious health problems; 63.7% perceived vaccine effective; higher education significantly associated with vaccine uptake (OR=1.80, p<0.001) | Positive attitudes but barriers include cost (22.5%) and lack of awareness (13.7%); healthcare provider recommendation key influencer (73.8%) |
| Abuzoor et al. (2025) [86] | Qualitative (semi-structured interviews) | 20 parents | Kuwait | Parents had variable knowledge; barriers: stigma, religion, societal influences, fear of complications, cost; perceived benefits and cues to action from healthcare practitioners can overcome barriers | HBM framework useful; correlation between sexual intercourse and HPV vaccine adds complexity; government and HCP recommendations crucial for acceptance |
| Bashari et al. (2025) [87] | Cost-effectiveness (Markov model) | 100,000 | Iran | Nine-valent vaccine most effective (98.4% CC reduction) but not cost-effective at current prices; ICERs: bivalent $33,179/QALY, quadrivalent $45,088/QALY, nine-valent $50,067/QALY; prices need 82-85% reduction to be cost-effective | No vaccination strategy cost-effective at current prices; high vaccine costs and low CC incidence are barriers |
| Onasoga et al. (2025) [88] | Cross-sectional | 380 | Nigeria | 68.2% had low knowledge; only 15% received HPV vaccine, 7.6% completed doses; predictors: age 20-25 (β=0.865, p=0.003), sexual activity (β=0.545, p<0.001), multiple partners, prior STIs, low knowledge level | Low knowledge and vaccine uptake; need for targeted interventions focusing on identified predictors to improve HPV vaccine coverage |
| Mohamed et al. (2025) [89] | Cross-sectional | 439 | Saudi Arabia | 58.8% accepted awareness level, 54.9% accepted knowledge level; 93.6% no Pap smear, 96.6% not vaccinated; medical students had significantly better knowledge than non-medical (p=0.028) | Female students lacked awareness, knowledge, and perception; need for culturally sensitive educational programs |
| Neisi et al. (2025) [90] | Case-control | 50 HPV-positive + 20 controls | Iran | Significant upregulation in HPV-positive samples: PI3K (24.59-fold, p<0.036), MALAT1 (9.75-fold, p<0.005), | PI3K, MALAT1, and H19 are promising biomarkers and therapeutic targets; |

| | | | | | |
|---|---|---|---|---|---|
| | | | | LINC00460 (1.15-fold, p<0.013), H19 (7.1-fold, p<0.018) | correlations between E6/E7 and lncRNAs observed but not statistically significant |
| Shabanpour et al. (2024) [91] | Cross-sectional | 130 | Iran | Three lineages found: A (7.73%), B (91.50%), D (0.77%); B1 sublineage dominant (91.50%); no statistically significant differences found between lineages and disease stages or amino acid changes | B1 sublineage dominant in Iran; further studies with larger sample sizes needed to assess pathogenicity risk of HPV58 lineages |
| Mobini Kesheh et al. (2022) [92] | Cross-sectional | 56 | Iran | HPV16 variants: D1 (81.58%), A4 (13.16%), A1 (2.63%), C1 (2.63%); HPV18 variants: A3 (92.85%), A4 (7.15%); most mutations affected YY1, TFIID, Oct-2, NF-1, c-Fos, and MBF1 binding sites | D1 and A3 dominant sublineages; mutations in transcription factor binding sites may affect viral oncogenic activity; women infected with these variants need longitudinal studies |
| Larijani et al. (2022) [93] | Cross-sectional | 43 | Iran | All samples belonged to lineage A; sublineages: A4 (69.7%), A1 (18.6%), A3 (11.6%); A2 and A5 not detected; no significant association between CIN and variants (p<0.05) | A4 sublineage dominant in Iran; lineage A identified in Iranian females; epidemiological data important for diagnostic tools and vaccine policy |
| Jalali-Alhosseini et al. (2024) [94] | Case-control | 64 | Iran | Lineages: A (61%), B (39%); A1 dominant among A lineage; no association between lineages and disease stage (p>0.05); amino acid K93R most common (14.1%) | A lineage and sublineage A1 common in Iran; further studies needed to estimate pathogenicity risk of HPV52 lineages |
| Taherkhani et al. (2025) [95] | Cross-sectional | 71 | Iran | HPV66: B (77.5%), A (22.5%); B2 dominant (64.8%). HPV68: C (66.1%), A (25.4%), D/E (6.8%), F (1.7%); C1 dominant (62.7%). No significant differences between lineages and histology/cytology status | B2 sublineage of HPV66 and C1 sublineage of HPV68 dominant in Iran; need for larger studies to elucidate pathogenicity risk |
| Al-Awadhi et al. (2019) [96] | Cross-sectional | 167 | Kuwait | Nine HPV genotypes detected; HPV27b most common (46.7%), followed by HPV57c (26.3%), HPV1a (9.6%), HPV2 (7.8%); single infection 93.4%, multiple 6.6% | α genus HPV types detected in 82% of patients; HPV27b and HPV57c most common in cutaneous warts; wart size significant feature of wart location |
| Koksal et al. (2021) [97] | Cross-sectional | 106 | Turkey | HPV DNA positive in 24.5%: HPV16 (84.6%), HPV18, HPV45, HPV82; p16INK4A positive in 18/106 (17%); concordance between HPV DNA and p16 91.2%; significantly higher HPV positivity in non-smokers (p=0.011) and patients <60 years (p=0.024) | HPV16 contributes to HNSCC development; HPV infection accounts for ~25% of HNSCC in Istanbul; smoking and age significantly associated with HPV positivity |
| Elhamady et al. (2025) [98] | Cross-sectional | 150 | Egypt | Genital warts cause significant sexual dysfunction: 10.7% severe ED, 15.3% | Genital warts significantly impair male sexual function and quality of life; need for |

| | | | | | |
|---|---|---|---|---|---|
| | | | | moderate, 29.3% mild; DLQI: 36.7% moderate impact, 26.7% very large impact; duration and size of warts, smoking significant predictors of dysfunction | routine sexual health assessments and psychological support |
| Morhason-Bello et al. (2025) [99] | Cross-sectional (secondary analysis) | 941 | Nigeria | Oral hrHPV: FGP 10.5%, FSW 14.9%, males 3.6% (p<0.001); Anal hrHPV: FGP 39.3%, FSW 60.8%, males 6.7% (p<0.001); Males had significantly lower odds of anal hrHPV (aOR=0.05, 95% CI:0.03-0.08) | Females had significantly higher oral and anal hrHPV prevalence than males; HIV status strongly associated with hrHPV at all sites; sex-specific prevention strategies needed |
| El-Sheikh et al. (2021) [100] | Cross-sectional / Observational | 72 | Egypt | HR-HPV DNA detected in 22.2% of BC cases (87.5% HPV-16), but 0% of BBL. Risk factors: low education, passive smoking, lack of physical activity, family history of cancer, and oral contraception use. | HR-HPV infection may be implicated in the development of some types of BC in Egyptian women. Identification of local risk factors can support practical prevention strategies for BC in Egypt. |
| Ceasar et al. (2025) [101] | Cross-sectional (Survey) | 460 | USA | Only 46% of ATs scored above 70% on knowledge. Those with prior training had significantly more knowledge (77.5% vs. 64%). ATs perceived a responsibility to educate stakeholders. | ATs are well-positioned to educate about HPV, but knowledge levels are low. Further education and training are required for ATs to be effectively involved in HPV prevention. |
| Kottaridi et al. (2022) [102] | Observational (Gene sequencing) | 40 | Greece | The T350G variation in the HPV16 E6 gene prevailed in 82.5% (33/40) of isolates. 85% of sequences clustered within the European variant branch. | The T350G variation may indicate a possible impact of this variant in the pathomechanisms of oropharyngeal cancer. Further studies are needed to explain its impact. |
| Yahyapour et al. (2018) [103] | Case-control | 168 | Iran | HPV in 27% ESCC vs. 41.2% non-neoplastic (p=0.066), EBV in 10% vs. 4.4% (p=0.143), MCPyV in 30% vs. 35.3% (p=0.471). No significant differences. | The study weakens the hypothesis of the pathogenic role of HPV, EBV, and MCPyV in esophageal malignant transformation. |
| Fakhraei et al. (2016) [104] | Cross-sectional | 100 | Iran | HPV DNA was detected in 5% (5/100) of samples. HPV-16 (3 cases), HPV-18 (1 case), and HPV-45 (1 case). | An association between HPV infection and gastric carcinoma in subjects from Northern Iran was not identified. |
| Elagali et al. (2021) [105] | Cross-sectional | 150 | Sudan | HPV DNA in 13/150 (8.67%) samples (HPV-16, 58, 18, 11). No significant association between HPV genotypes and BC grading, histological types, P53/RB mutations, or ER/PR expression. Significant association between P53/RB mutations and histological types. | A statistically insignificant association was observed between HPV and BC. P53 and RB gene mutations were significantly associated with different BC histological types. |

| | | | | | |
|---|---|---|---|---|---|
| Dialameh et al. (2021) [106] | Cross-sectional | 82 | Iran | HPV PCR positivity was significantly higher in PTC (13.4%) compared to benign nodules (3.8%) and normal tissue (0%) (P=0.015). | There was a significant association between PTC and HPV positivity. Further studies are required to determine the cause and effect. |
| Alsbeih et al. (2019) [107] | Observational | 285 | Saudi Arabia | HPV-DNA was positive in 3.5% (10/285) overall (21% oropharyngeal, 2% oral cavity). p16 was positive in 42% of 50 tested cases. HPV/p16 positivity was associated with better survival (p=0.05). | HPV-DNA prevalence was significantly lower than worldwide estimates. HPV/p16 status was an important determinant of patients' survival, helping distinguish favorable from unfavorable tumors. |
| Fernandes et al. (2022) [108] | Review | N/A | MENA region | Pooled HPV prevalence in cervical cancer is 81% in MENA; HPV16 and HPV18 are most common. | Limited vaccination and screening in MENA; urgent need for national HPV programs and larger studies. |
| Salhi et al. (2025) [109] | Retrospective | 48 (40 with DNA) | Palestine | HPV prevalence in cervical cancer tissues was 82.5%; HPV16 (32.5%) and HPV18 (10%) most frequent. | High prevalence of high-risk HPV underscores the need for nationwide vaccination and screening programs. |
| Keikha et al. (2025) [110] | Descriptive-analytical cross-sectional | 320 | Iran | Mean knowledge score was very low (4.39±5.01); attitude was moderate (18.57±2.80). | Educational interventions are necessary, especially in vulnerable provinces with low literacy and early marriage. |
| Nouh et al. (2026) [111] | Descriptive cross-sectional | 530 nursing students | Egypt | Nursing students had low knowledge about HPV infection (36.6%) and vaccination (45.1%) with negative attitudes | None of the nursing students (0%) had received HPV vaccination |
| Haddadi et al. (2026) [112] | Cross-sectional | 8,158 females | Iran | HPV vaccination coverage was 0.85% and cervical cancer screening participation was 39.4% | HPV vaccination coverage was far below WHO CCEI target of 90% |
| Akhtar et al. (2026) [113] | Cross-sectional | 1,341 female schoolteachers | Kuwait | 60% were unaware of HPV causal role in cervical cancer; 98.1% were HPV unvaccinated | 88% were unaware of HPV vaccine availability |
| Hossein zadeh et al. S (2026) [114] | Cross-sectional | 214 (121 male, 93 female) | Iran | Overall HPV prevalence was 28%; 43.4% had high-risk genotypes | HPV16 and HPV31 were most common high-risk genotypes in both genders |
| Finney et al. (2026) [115] | Retrospective cross-sectional | 229 women | UAE | HPV prevalence was 42%; HR53, 16, 68, 66, 31 were predominant genotypes | 21% of NILM samples tested positive for HPV |
| Cassani et al. (2024) [116] | Systematic review & meta-analysis | 47 studies, 8,321 subjects | Multiple countries | Multiple HR-HPV infections were more prevalent in HIV+ women (39.1%) vs HIV-negative (21.6%) | Multiple HR-HPV increased risk of HSIL persistence/recurrence (OR:1.38, 95% CI:1.08-1.75) |
| Razavi Nikoo et al. (2026) [117] | Cross-sectional | 1004 | Iran | HPV prevalence 38.94% with HPV-6, -11, -16, -62, -81 most common | Highest infection in 25-34 age group; HPV-16 prevalence 4.48% underscores vaccination importance; provides baseline for screening programs |
| Habibpour et al. (2026) [118] | Cross-sectional | 395 | Iran | HPV-positive women had significantly lower sexual quality of life | β=-4.39, CI=-7.94,-0.84; self-worthlessness and sexual suppression subscales significantly decreased; female occupation |

| | | | | | |
|---|---|---|---|---|---|
| | | | | | (employed) associated with lower sexual quality of life. |
| Chellat et al. (2026) [119] | Cross-sectional | 997 | Morocco | HPV self-sampling feasible and highly acceptable; 13% opted for self-sampling | 92.2% found procedure easy; all reported comfort using cytobrush; diagnostic performance comparable to clinician-collected samples; low uptake in rural settings highlights need for education |
| Karami et al. (2026) [120] | Cross-sectional | 211 | Iran | Total HPV prevalence 6.63%; HPV16 and HPV11 accounted for 28.50% equally | Multiple HPV infections seen in 21.40%; significant association between HPV positivity and cellular changes; ASC-US most common lesion (64.3%) |
| Eslamloueian et al. (2026) [121] | Case-control | 272 (136 cases, 136 controls) | Iran | Women with genital warts experienced significantly higher sexual distress (26.16±13.97 vs 8.50±6.42) | No significant difference in sexual avoidance; 85.29% of case group had sexual distress disorder; dyspareunia (P=0.003), higher age at first intercourse (P=0.004) associated with higher sexual distress |
| Karami et al. (2025) [122] | Cross-sectional | 125 | Iran | HPV-16 lineages A (32.8%), C (0.8%), D (66.4%) identified; lineage D dominant | Integrated form more prevalent in ICC patients (44.6%) than CIN I-III (23.1%) (P=0.000048); association between lineage D and integrated form when stratified by histology (P=0.0028); no significant association between overall lineages and integration status (P=0.85) |


1. Alhamlan, F.S.; Alfageeh, M.B.; Al Mushait, M.A.; Al-Badawi, I.A.; Al-Ahdal, M.N. Human Papillomavirus-Associated Cancers. *Adv Exp Med Biol* **2021**, *1313*, 1-14, doi:10.1007/978-3-030-67452-6_1.
2. Shalchimanesh, Z.; Ghane, M.; Kalantar, E. Prevalence of Human Papillomavirus Genotypes in Tehran, Iran. *J Res Health Sci* **2022**, *22*, e00553, doi:10.34172/jrhs.2022.88.
3. Aliabadi, N.; Hosseini, M.; Doustfatemeh, S.; Marandi, N.H.; Joukar, M.; Faramarzi, H.; Asmarian, N.; Namazi, N. Human Papillomavirus (HPV) and Infertility: A Cross-Sectional Study Exploring Its Prevalence and Effects in Iranian Couples. *Health Sci Rep* **2025**, *8*, e71624, doi:10.1002/hsr2.71624.
4. Moradi, P.; Farahani, A.; Mohajeri, P.; Izadi, B.; Abiri, R.; Alvandi, A.; Rezaei, M. Prevalence of human papillomavirus (HPV) genotypes in patients with clinical symptoms in Kermanshah, western Iran: a cross-sectional study. *BMC Infect Dis* **2025**, *25*, 226, doi:10.1186/s12879-025-10563-9.
5. Mojarrad, S.; Najmafshar, M.; Jahromi, Z.K.; Salahi Ardekani, O.; Shahraki, H.R.; Jalvand, M.; Sana, F.A. Prevalence and genotype distribution of human papillomavirus in individuals referred to a laboratory in Urmia, Iran. *Infect Agent Cancer* **2025**, *20*, 13, doi:10.1186/s13027-025-00636-4.
6. Küçükyurt, A.K.; Atakul, N.; Kolcu, S.A.; Özcan, A.J.; Abdullazade, N. Is age a determinant in cervical cancer screening in women aged 18 to 29?: An observational study. *Medicine (Baltimore)* **2025**, *104*, e42765, doi:10.1097/md.0000000000042765.

7. Aygar İ, S.; Yavuz, M.T. Comprehensive Molecular Epidemiology of HPV in a High-Volume Tertiary Center. *Infect Dis Clin Microbiol* **2025**, *7*, 301-309, doi:10.36519/idcm.2025.659.
8. El-Daly, M.M.; Faizo, A.A.; Madkhali, S.A.; Aldardeir, N.F.; Badroon, N.A.; Uthman, N.A.; Alqawas, R.T.; Mousa, A.A.; Turkistani, S.A.; Azhar, E.I. Study of the Prevalence of Human Papillomavirus Genotypes in Jeddah, Saudi Arabia. *J Epidemiol Glob Health* **2025**, *15*, 1, doi:10.1007/s44197-025-00340-x.
9. Tanik, E.B.; Bakir, A.; Turkmenoglu, T.T.; Erdem, G. Distribution of High-risk Human Papillomavirus Genotypes in Cervical Smear Samples and Evaluation of ASC-US, LSIL, and HSIL Results. *J Cytol* **2024**, *42*, 37-42, doi:10.4103/joc.joc_70_24.
10. Sadeghi, F.; Mostaghimi, T.; Taheri, M.; Yazdani, S.; Javadian, M.; Ranaee, M.; Ghorbani, H.; Bouzari, Z.; Sadeghi, F. Investigating the role of Epstein-Barr virus and human papillomavirus types 16 and 18 co-infections in cervical disease of Iranian women. *Front Oncol* **2024**, *14*, 1331862, doi:10.3389/fonc.2024.1331862.
11. Letafati, A.; Noroozi, M.; Fallah, T.; Farahani, A.V.; Nasiri, M.M.B.; Pourmoein, H.; Sadeghi, Z.; Ardekani, O.S.; Heshmatipour, K.; Nodeh, S.Y.; et al. Distribution of HPV genotypes in Mashhad, Iran: insights from a 2022-2023 study. *Virol J* **2024**, *21*, 248, doi:10.1186/s12985-024-02518-4.
12. Letafati, A.; Motlaghzadeh, S.; Ardekani, O.S.; Memarpour, B.; Seyedi, S.; Bahari, M.; Farahani, A.V.; Khoshravan, A.; Sarrafzadeh, S.; Vasmehjani, A.A.; et al. Uncommon high distribution of HPV-16, HPV-54, and HPV-56 in female referred to a laboratory in Karaj, Iran: indications of a paradigm shift in HPV genotypes? *Virol J* **2024**, *21*, 182, doi:10.1186/s12985-024-02457-0.
13. Gholamzad, A.; Khakpour, N.; Hashemi, M.; Gholamzad, M. Prevalence of high and low risk HPV genotypes among vaccinated and non-vaccinated people in Tehran. *Virol J* **2024**, *21*, 9, doi:10.1186/s12985-023-02270-1.
14. Etemad, S.; Asghari Baghalan, A.M.; Naeimi, B.; Mehrzad, S.; Adib Amin, S.; Soudyab, M.; Jafarzadeh Esfehani, R. Prevalence of Human Papillomavirus (HPV) Infection and its Association With Pap Smear Findings Among Women Attending a Gynecology Clinic in Khorasan Razavi-Iran. *J Res Health Sci* **2024**, *24*, e00629, doi:10.34172/jrhs.2024.164.
15. Haddadi, M.; Atefmehr, L.; Motlaghzadeh, S.; Hejami, F.; Elyasi, F.S.; Zafarian, N.; Taghiabadi, Z.; Aboofazeli, A.; Yarahmady, H.; Modaresi, P.; et al. Prevailing of HPV-16 and 52 genotype in 2022-2023 in Sanandaj, Iran. *Virol J* **2024**, *21*, 106, doi:10.1186/s12985-024-02373-3.
16. Dura, M.C.; Aktürk, H.; Aslan, Ö.; Yıldız, Ş.; Kefeli, M.; Çelik, H. Human Papillomavirus Infection (HPV) Prevalence in the Black Sea Region of Turkey: Primary HPV Screening for Cervical Cancer. *Cureus* **2024**, *16*, e52615, doi:10.7759/cureus.52615.
17. Pebdeni, P.H.; Saffari, F.; Mollaei, H.R.; Mirshekari, T.R.; Sadat, R.H.; Habibzadeh, V.; Saeed, L.; Soodejani, M.T.; Ahmadrajabi, R. Increased Risk of Infertility in Women Infected with Human Papillomavirus. *J Reprod Infertil* **2023**, *24*, 188-197, doi:10.18502/jri.v24i3.13275.
18. Bakhshani, A.; Ganjali, R.; Tabatabaeizadeh, S.E. Prevalence of Human Papillomavirus (HPV) Genotypes among Women During 2015-2020 in Mashhad, Iran. *Arch Iran Med* **2023**, *26*, 419-426, doi:10.34172/aim.2023.64.
19. Taskin, M.H.; Nursal, A.F.; Oruc, M.A.; Kariptas, E. Genotype Distribution and Prevalence of High-Risk Human Papillomavirus Infection among Women in Samsun Province of Turkey. *Asian Pac J Cancer Prev* **2022**, *23*, 2477-2482, doi:10.31557/apjcp.2022.23.7.2477.
20. Tekkesın, N.; Goktas, S.; Alkis, V.; Koc, S.; Gurbuz, T.; Goktas, P. Analysis of the Prevalence and Quantification of viral load of different human papillomavirus types in Turkish women population. *Asian Pac J Cancer Prev* **2022**, *23*, 4347-4355, doi:10.31557/apjcp.2022.23.12.4347.

21. Saeed, N.K.; Alshaikh, S.; Al-Beltagi, M. Comparative study of pap smear and polymerase chain reaction tests for human papillomavirus screening in Bahrain. *East Mediterr Health J* **2025**, *31*, 453-462, doi:10.26719/2025.31.7.453.
22. Sait, K.H.; Anfinan, N.M.; Sait, H.K.; Basalamah, H.A. Human papillomavirus prevalence and dynamics: Insights from a 5-year population-based study in Jeddah, Kingdom of Saudi Arabia. *Saudi Med J* **2024**, *45*, 252-260, doi:10.15537/smj.2024.45.3.20230824.
23. Dastjerd, N.T.; Abbasian, L.; Mohebbi, S.R.; Banaem, L.M.; Ashtiani, M.F.; Rostamafshar, Z.; Soleimanjahi, H.; Sarshari, B. Prevalence and distribution of human papillomavirus genotypes in women living with HIV: a cross-sectional study in Iran. *BMC Infect Dis* **2025**, *25*, 1471, doi:10.1186/s12879-025-11805-6.
24. Banjer, H.J.; Tayeb, H.H.; Alzahrani, K.J.; Alsufiani, S.M.; Alrowaihi, J.M. Cervical cancer risk and high-risk HPV types distribution in Saudi Arabia: Trends from a national laboratory-based study. *Saudi Med J* **2025**, *46*, 992-999, doi:10.15537/smj.2025.46.9.20250266.
25. El-Daly, M.M.; Faizo, A.A.; Madkhali, S.A.; Badroon, N.A.; Othman, N.A.; Alandijany, T.A.; Moria, A.; Turkistani, S.A.; El-Kafrawy, S.A.; Azhar, E.I. Human papillomavirus genotypes in Jeddah, Saudi Arabia: prevalence and public health implications. *Infect Agent Cancer* **2025**, *20*, 79, doi:10.1186/s13027-025-00705-8.
26. El-Wakil, D.M.; Shaker, O.G.; Rashwan, A.; Elesawy, Y.F.; Samir, N. High-risk human papillomavirus infection and cervical cytopathology: relationship with cervical nitric oxide levels. *Virol J* **2024**, *21*, 173, doi:10.1186/s12985-024-02435-6.
27. Abdoudaim, M.S.; Bélec, L.; Ahmed, M.; Baba, N.D.M.; Mboumba Bouassa, R.S.; Abdellahi, M.V.M. Human Papillomavirus Viral Load as Triage Biomarker for High-Grade Cervical Lesions and Invasive Cervical Carcinoma: A Cross-Sectional Study. *Health Sci Rep* **2025**, *8*, e71524, doi:10.1002/hsr2.71524.
28. Ashry, M.; Shawky, S.; Mounir, Z.; Fathy, F.; Elsayed, H.; Kamal, W.; Hassany, M. Prevalence and risk factors of human papilloma virus infection among women living with HIV, Egypt, a cross sectional study. *BMC Public Health* **2024**, *24*, 1821, doi:10.1186/s12889-024-19240-z.
29. Mahsa, N.; Rasool, H.; Pardis, K.S.; Adel, H. Genotyping of human papillomaviruses in patients with Recurrent Respiratory Papillomatosis in Firouzgar Hospital, Tehran, Iran. *Arch Razi Inst* **2025**, *80*, 887-896, doi:10.32592/ari.2025.80.4.887.
30. Sadeghi, Z.; Jazayeri, S.M.; Pirkooh, A.A.; Foroushani, A.R.; Yarandi, F.; Alebouyeh, M.; Bakhtiari, R. Cell cycle gene expression and microbial profiles associated with HPV related cervical neoplasia and cervical microbiome shifts. *Sci Rep* **2026**, *16*, doi:10.1038/s41598-026-36885-5.
31. Aslanimehr, M.; Nemati, S.; Sadeghi, H.; Samiee-Rad, F.; Ghafari, S.; Naserpour-Farivar, T. High-risk HPV genotypes in women with abnormal cytology: a 12-year retrospective study. *Infect Agent Cancer* **2025**, *20*, 34, doi:10.1186/s13027-025-00664-0.
32. Soleimani-Jelodar, R.; Arashkia, A.; Shoja, Z.; Sharifian, K.; Akhavan, S.; Yarandi, F.; Jalilvand, S. Type-specific human papillomavirus prevalence in women referred for colposcopy in Tehran. *Iran J Microbiol* **2024**, *16*, 421-427, doi:10.18502/ijm.v16i3.15799.
33. Moeinzadeh, M.; Kheirkhah, B.; Amini, K.; Pouryasin, A. Classification and identification of human papillomavirus based on its prevalence and development of cervical lesion among Iranian women. *Bioimpacts* **2020**, *10*, 235-242, doi:10.34172/bi.2020.30.
34. Letafati, A.; Farahani, A.V.; Nasiri, M.M.B.; Pourmoein, H.; Ardekani, O.S.; Ahoodashty, H.; Hashemi-Soteh, M.B.; Dadgar, A.; Behshood, P.; Azhar, I.R.; et al. High prevalence of HPV-56 and HPV-39 in Sari, Iran: an analysis of genotype distribution. *Virol J* **2024**, *21*, 227, doi:10.1186/s12985-024-02496-7.

35. Yerim, S.A.; Khazraji, Y.C.; Bekkali, R.; Bennai, M.; Bahra, N.; Chaoui, I.; Chellat, F.Z.; Gaizi, Z.; Tachfouti, N.; Benabdellah, A.; et al. Evaluating the performance of the Xpert HPV assay in detecting HPV positive cases in Morocco. *Tumour Virus Res* **2025**, *19*, 200318, doi:10.1016/j.tvr.2025.200318.
36. Çubuk, F.; Yıldız, M.; Balcı, F.; İrgin, A.; Aşkın, S.; Sağtaş, E.; Kaygusuz, S. A new approach for detecting HPV DNA in cervical swabs: comparison of nucleic acid extraction with direct PCR. *Virol J* **2025**, *22*, 8, doi:10.1186/s12985-024-02601-w.
37. Ben Taher, Y.; Ben Rekaya, M.; Romdhane, E.; Miladi, B.; Bel Hadj Kacem, L.; Med H'mayada, A.; Ben Mansour, N.; Sassi, F.; Chelbi, E.; Tangour, M.; et al. HPV testing in cervical cancer formalin-fixed paraffin embedded tissues: Reliability of the Xpert HPV test and high-risk HPV genotypes distribution in Tunisia. *PLoS One* **2025**, *20*, e0333600, doi:10.1371/journal.pone.0333600.
38. Guzelbag, B.; Uzuner, E.G.; Selcuk, M.; Ermin, S.; Ozgun Selcuk, C.G.; Cim, N. Twelve-year trends in cervical cytology screening: a retrospective analysis of 83,148 smears from a Turkish tertiary care center. *Front Public Health* **2026**, *14*, 1818289, doi:10.3389/fpubh.2026.1818289.
39. Tahir, M.; Mohammed, D.; Sadiq, C. Prevalence and genotypic characterization of human papillomavirus in Iraqi women: a retrospective study of 2,193 cases. *Virol J* **2025**, *22*, 394, doi:10.1186/s12985-025-03021-0.
40. Shahi, M.; Shafaei, A.; Ghodsi, M.; Esfehani, R.J.; Moradi Marjaneh, M. HPV Genotype Trends in Iran: Necessity for a Reevaluation of Prevention Strategies. *Trop Med Infect Dis* **2025**, *10*, doi:10.3390/tropicalmed10040100.
41. Sadeghi, Z.; Aboofazeli, A.; Sarrafzadeh, S.; Tayebi, N.; Tajdini, R.; Yarandi, F.; Jazayeri, S.M. Investigating the link between HPV genotypes and cervical abnormality incidence in women with HPV infections: insights from a leading referral centre. *Virol J* **2025**, *22*, 264, doi:10.1186/s12985-025-02858-9.
42. Nikkhoo, B.; Mahmoodian, S.; Bahrami, P.; Daraee, A.; Soleimani, F.; Zamani, F.; Nasseri, S.; Rahmani, K. Prevalence and genotype distribution of human papillomavirus infection among Kurdish people, Iran. *SAGE Open Med* **2025**, *13*, 20503121251320522, doi:10.1177/20503121251320522.
43. Kılıç, H.; İlhan Çelik, Ö. Histopathological Correlation of Cases with Epithelial Cell Abnormality Detected in Cervicovaginal Smear Screenings of Turkish Women and Their Association with HPV Subtypes. *Int J Gen Med* **2025**, *18*, 3871-3879, doi:10.2147/ijgm.S546538.
44. Alci, A.; Yalcin, N.; Gokkaya, M.; Sari, G.E.; Turkmenoglu, H.R.; Ureyen, I.; Toptas, T. Does Co-Infection with HPV 16 Have a Worse Effect on Cervical Pathology than HPV 16 Alone? *Sisli Etfal Hastan Tip Bul* **2025**, *59*, 338-344, doi:10.14744/semb.2025.34682.
45. Gunes, A.C.; Ozgul, N.; Turkyılmaz, M.; Kara, F.; Unlu, F.; Ayhan, A.; Gultekin, M. Evaluation of colposcopy after the addition of human papillomavirus testing to the Turkish cervical cancer screening program. *Cancer Med* **2023**, *12*, 21751-21760, doi:10.1002/cam4.6740.
46. Shabanpour, M.; Taherkhani, S.; Shoja, Z.; Eftekhari, M.; Jalilvand, S. Lineage analysis of human papillomavirus types 33 and 35 based on E6 gene in cervical samples from Tehran, Iran. *Sci Rep* **2025**, *15*, 13736, doi:10.1038/s41598-025-97080-6.
47. Samaria, D.; Tallutondok, E.B.; Simanjorang, C.; Imanuel, I. Comparison of Self-Efficacy and Knowledge of Human Papillomavirus Vaccination among Various Types of Decision Makers in Indonesia: A Cross-Sectional Study. *Int J Community Based Nurs Midwifery* **2025**, *13*, 28-39, doi:10.30476/ijcbnm.2024.101802.2448.

48. Falahi, S.; Arvaneh, K.; Nourmohammadi, H.; Abdoli, A.; Bozorgomid, A.; Kenarkoohi, A. Prevalence and genotyping of human papillomavirus DNA in peripheral blood mononuclear cells from blood donors in Iran. *New Microbes New Infect* **2025**, *65*, 101593, doi:10.1016/j.nmni.2025.101593.
49. Abi Aad, Y.; Ballouz, T.; Faysal, H.; Mahfouz, R.; Shabb, N.; Sader, G.; El-Helou, R.; Doughan, S.; Rizk, N.A. Prevalence of human papilloma virus infection and anal dysplasia among men with HIV in Lebanon: a cross-sectional study. *HIV Res Clin Pract* **2024**, *25*, 2425548, doi:10.1080/25787489.2024.2425548.
50. Khezeli, Z.; Shoja, Z.; Kaffashian, M.; Soleimani-Jelodar, R.; Jalilvand, S. The Lineage and Sublineage Investigation of Human Papillomavirus Type 16 in Tehran, Iran, During 2022-2023: A Cross-Sectional Study. *Health Sci Rep* **2025**, *8*, e70680, doi:10.1002/hsr2.70680.
51. Shafaei, A.; Ali Akbar Shamsian, S.; Ghodsi, M.; Sadabadi, F.; Shahi, M. Influence of sexually transmitted infections on the cervical cytological abnormalities among Iranian women: A cross-sectional study. *Int J Reprod Biomed* **2023**, *21*, 491-498, doi:10.18502/ijrm.v21i6.13636.
52. Mortazavi, S.M.; Tarinjoo, A.; Dastani, S.; Niyazpour, M.; Dahaghin, S.; Mirnejad, R. Molecular Detection of Sexually Transmitted Infections in Women with and without Human Papillomaviruses Infection Who Referred to Tehran West Hospitals in Iran. *Rep Biochem Mol Biol* **2021**, *10*, 387-395, doi:10.52547/rbmb.10.3.387.
53. de Oliveira, C.M.; Rangeiro, R.; Osman, N.; Baker, E.; Neves, A.; Mariano, A.A.N.; Tivir, G.; Thomas, J.P.; Carns, J.; Andrade, V.; et al. Evaluation of hpv risk groups among women enrolled in the mulher cervical cancer screening study in Mozambique. *Infect Agent Cancer* **2025**, *20*, 24, doi:10.1186/s13027-025-00655-1.
54. Azadehrah, M.; Azadehrah, M.; Zeinali, F.; Nokhostin, F. Prevalence of Trichomonas Infection in Relation to Human Papillomavirus (HPV) in Pap Smear Samples of Female Patients Referred to Shahid Sadoughi Hospital, Yazd (Iran). *Cureus* **2024**, *16*, e57701, doi:10.7759/cureus.57701.
55. Sadati, M.S.; Jahangiri, S.; Azizi, A.; Parvar, S.Y.; Jahromi, M.A.; Dehghan, Y. Prevalence and Distribution of HPV Genotypes in Patients With Genital Warts: A Cross-Sectional Study From South of Iran. *Health Sci Rep* **2025**, *8*, e71345, doi:10.1002/hsr2.71345.
56. Zaky, M.S.; Obaid, Z.M.; Youssef, A.M.; Elmenawy, A.D.; Elsaie, M.L. Sexual dysfunction in women with genital warts: a cross-sectional study. *BMC Womens Health* **2025**, *25*, 188, doi:10.1186/s12905-025-03691-6.
57. Farhadi, A.; Abuei, H.; Okhovat, M.A.; Geramizadeh, B.; Behzad-Behbahani, A.; Chong, P.P.; Nikouyan, N.; Namdari, S. Type distribution of human papillomaviruses in ThinPrep cytology samples and HPV16/18 E6 gene variations in FFPE cervical cancer specimens in Fars province, Iran. *Cancer Cell Int* **2023**, *23*, 166, doi:10.1186/s12935-023-03011-8.
58. Alwadai, M.S.; Abdulghani, N. Assessment of knowledge and attitudes toward human papillomavirus and its vaccination among female nursing students at Umm Al-Qura University, Saudi Arabia. *Front Glob Womens Health* **2025**, *6*, 1669950, doi:10.3389/fgwh.2025.1669950.
59. Alshehri, M.A.; Fahim, W.A.; Alsaigh, R.R. The Association Between Parents' Knowledge About Human Papillomavirus and Their Intention to Vaccinate Their Daughters: A Cross-Sectional Study. *Cureus* **2023**, *15*, e48600, doi:10.7759/cureus.48600.
60. Solak, B.; Arslan, M. Evaluation of HPV and Related Cancer Awareness and Vaccination Attitudes Among Patients with Anogenital Warts: a Survey-Based Study. *J Community Health* **2025**, *50*, 560-567, doi:10.1007/s10900-025-01444-y.

61. Elmanzlawey, M.; Terra, M.; Baklola, M.; Ahmed, Y.; Abuelela, M.; Al-Kurd, I.N.; El-Gilany, A.H. Awareness, knowledge, and beliefs about Human Papillomavirus and its vaccine among Egyptian medical students: A cross-sectional national study. *PLoS One* **2025**, *20*, e0337411, doi:10.1371/journal.pone.0337411.
62. Alghamdi, R.; Alghamdi, M.; Farsi, N.; Alyafi, M.; Alaidaroos, S.; Almuwalad, N.; Alsaeedi, R.; Alshobaki, K.; Aljabri, R.; Aljadani, R.; et al. Knowledge of the human papilloma virus and acceptance of its vaccine among male and female young adults in Jeddah, Saudi Arabia: A cross-sectional study. *Hum Vaccin Immunother* **2025**, *21*, 2583609, doi:10.1080/21645515.2025.2583609.
63. Erbıyık, H.I.; Palalıoğlu, R.M. HPV Infection, HPV Vaccines and Cervical Cancer Awareness: A Multi-Centric Survey Study in Istanbul, Turkey. *Women Health* **2021**, *61*, 771-782, doi:10.1080/03630242.2021.1969612.
64. Cheema, S.; Abraham, A.; Maisonneuve, P.; Jithesh, A.; Chaabna, K.; Al Janahi, R.; Sarker, S.; Hussain, A.; Rao, S.; Lowenfels, A.B.; et al. HPV infection and vaccination: a cross-sectional study of knowledge, perception, and attitude to vaccine uptake among university students in Qatar. *BMC Public Health* **2024**, *24*, 2316, doi:10.1186/s12889-024-19792-0.
65. Al-Antary, N.; Siddiqui, R.F.; Gonzalez, K.; Nair, M.; Gilbert, M.; Fakhoury, L.; Vu, M.; Siddiqui, F.; Adjei Boakye, E. Awareness of HPV, HPV vaccine and HPV-associated cancers among Arab Americans. *Sci Rep* **2025**, *16*, 1897, doi:10.1038/s41598-025-31575-0.
66. Tehranian, A.; Vahid Dastjerdi, M.; Hatamian, S.; Ghahghaei-Nezamabadi, A. Sexual Function and Quality of Life in Iranian Women With Human Papillomavirus Infection. *J Family Reprod Health* **2024**, *18*, 154-159, doi:10.18502/jfrh.v18i3.16656.
67. Doğan, A.; Toker, M.S.; Gozdas, H.T.; Bal, T. Knowledge and Attitude of Turkish Male Health Care Workers Regarding Human Papillomavirus and Vaccination. *Am J Mens Health* **2024**, *18*, 15579883241304574, doi:10.1177/15579883241304574.
68. Alsulami, F.T. Exploring the Impact of Knowledge about the Human Papillomavirus and Its Vaccine on Perceived Benefits and Barriers to Human Papillomavirus Vaccination among Adults in the Western Region of Saudi Arabia. *Healthcare (Basel)* **2024**, *12*, doi:10.3390/healthcare12141451.
69. Alherz, F.A.; Alamri, A.A.; Aljbreen, A.; Alwallan, N. Knowledge of cervical cancer, human papillomavirus (HPV), and acceptance of the HPV vaccine among parents of daughters in Riyadh, Saudi Arabia. *J Infect Public Health* **2024**, *17*, 789-794, doi:10.1016/j.jiph.2024.03.014.
70. Abdoudaim, M.S.; Mohamed Abdellahi, M.V.; Mohamed Baba, N.D.; Mboumba Bouassa, R.S.; Ahmed, M.; Bélec, L. Human Papillomavirus Genotypes Distribution in High-Grade Cervical Lesions and Invasive Cervical Carcinoma in Women Living in Mauritania: Implications for Cervical Cancer Prevention and HPV Prophylactic Vaccination. *Diagnostics (Basel)* **2024**, *14*, doi:10.3390/diagnostics14171986.
71. Shakurnia, A.; Salehpoor, F.; Ghafourian, M.; Nashibi, R. Knowledge and attitudes toward HPV, cervical cancer and HPV vaccine among healthcare providers in Ahvaz, Southwest Iran. *Infect Agent Cancer* **2025**, *20*, 44, doi:10.1186/s13027-025-00669-9.
72. Yörük, S.; Açıkgöz, A.; Ergör, G. Determination of knowledge levels, attitude and behaviors of female university students concerning cervical cancer, human papiloma virus and its vaccine. *BMC Womens Health* **2016**, *16*, 51, doi:10.1186/s12905-016-0330-6.
73. Azzi, A.; Al-Dhelaan, R.; Al-Fahd, A.; Almukhalafi, O.; Al-Tayar, N.; Abutheeb, A.; Bahmid, M. Assessment of awareness level among women about cervical smears, human papillomavirus (HPV), and HPV vaccine in Saudi Arabia. *Sci Rep* **2025**, *15*, 44171, doi:10.1038/s41598-025-27814-z.

74. Mahmoud, I.; Al Eid, M.M.A.; Mohamed, M.A.; Aladwani, A.J.; El Amin, N.E.M. Human papillomavirus vaccination and Pap test uptake, awareness, and barriers among young adults in Gulf Cooperation Council countries: A comparative cross-sectional survey. *J Infect Public Health* **2024**, *17*, 102525, doi:10.1016/j.jiph.2024.102525.
75. Gari, A.; Ghazzawi, M.A.; Ghazzawi, S.A.; Alharthi, S.M.; Yanksar, E.A.; Almontashri, R.M.; Batarfi, R.; Kinkar, L.I.; Baradwan, S. Knowledge about cervical cancer risk factors and human papilloma virus vaccine among Saudi women of childbearing age: A community-based cross-sectional study from Saudi Arabia. *Vaccine X* **2023**, *15*, 100361, doi:10.1016/j.jvacx.2023.100361.
76. Varer Akpinar, C.; Alanya Tosun, S. Knowledge and perceptions regarding Human Papillomavirus (HPV) and willingness to receive HPV vaccination among university students in a north-eastern city in Turkey. *BMC Womens Health* **2023**, *23*, 299, doi:10.1186/s12905-023-02455-4.
77. Tobaiqy, M.A.; Mehdar, S.A.; Altayeb, T.I.; Saad, T.M.; Alqutub, S.T. Parental knowledge, views, and perceptions of human papilloma virus infection and vaccination-cross-sectional descriptive study. *J Family Med Prim Care* **2023**, *12*, 556-560, doi:10.4103/jfmpc.jfmpc_1673_22.
78. Abdelhadi, L.M.; Aryan, F.S.; Alsabi, R.; Samhan, G.A.; Al-Qaaneh, A.M. Knowledge, Attitudes, and Practices Associated with Human Papillomavirus Vaccine Recommendation Among Healthcare Professionals: A Cross-Sectional Study. *Infect Dis Rep* **2025**, *17*, doi:10.3390/idr17050126.
79. Alghanmi, R.; Alkhalawi, E.; Albeladi, R.; Albeladi, S.; Alghamdi, M.; Fayoumi, A.; Nassif, R. Parental Knowledge and Acceptance of HPV Vaccine in Rabigh's School, Saudi Arabia. *Ann Glob Health* **2026**, *92*, 13, doi:10.5334/aogh.4866.
80. Taumberger, N.; Friko, I.; Der, V.M.; Ellis, L.B.; Shearer, A.M.; Bowden, S.J.; Kyrgiou, M.; Pan, T.L.; Lessiak, V.; Hofer, N.; et al. Implementing High-Risk Human Papillomavirus Self-Sampling for Cervical Cancer Screening in Ghana: A Study (CarciSCAN) Protocol. *Cancer Control* **2025**, *32*, 10732748251330698, doi:10.1177/10732748251330698.
81. Sadat Larijani, M.; Javadi, A.; Fahimzad, A.; Soleimani, R.; Tabatabaei, F.; Nejadeh, A.H.; Jamshidi, M.; Bavand, A.; Moradi, L.; Ashrafian, F.; et al. Prevalence of oral HPV infection and genotype distribution in Iranian children during the early ages of life. *Virol J* **2025**, *22*, 148, doi:10.1186/s12985-025-02767-x.
82. Kayar, İ.; Goc, G.; Cetin, F.; Birge, Ö. Impact of Smoking on Cervical Histopathological Changes in High-Risk HPV-Positive Women: A Matched Case-Control Study. *Medicina (Kaunas)* **2025**, *61*, doi:10.3390/medicina61020235.
83. Darvishali, M.H.; Nikkhah, H.; Rahavi, H.; Jalali, H.; Vakili Ghasem Abad, M.; Vajihinejad, M.; Farashahi-Yazd, E. HPV Genotypes and Survival Outcomes in Invasive Cervical Cancer: A Retrospective Molecular Analysis of FFPE Clinical Samples in an Iranian Cohort. *Int J Womens Health* **2026**, *18*, 589530, doi:10.2147/ijwh.S589530.
84. Alghalyini, B.; Zaidi, A.R.Z.; Meo, S.A.; Faroog, Z.; Rashid, M.; Alyousef, S.S.; Al-Bargi, Y.Y.; Albader, S.A.; Alharthi, S.A.A.; Almuhanna, H.A. Awareness and knowledge of human papillomavirus, vaccine acceptability and cervical cancer among college students in Saudi Arabia. *Hum Vaccin Immunother* **2024**, *20*, 2403844, doi:10.1080/21645515.2024.2403844.
85. Faqeeh, H.; Alsulayyim, R.; Assiri, K.; Alqhatani, M.; Zanquti, A.; Hakami, W.; Amri, K.; Akaram, S. Perceptions, Attitudes, and Barriers to Human Papillomavirus Vaccination Among Residents in Saudi Arabia: A Cross-Sectional Study. *Cureus* **2024**, *16*, e57646, doi:10.7759/cureus.57646.

86. Abuzoor, A.; Jabin, M.S.R.; Eshareturi, C.; Nesbitt, R.; Jonker, C. Using the Health Belief Model to Examine Parental Knowledge and Health Beliefs About Human Papilloma Virus (HPV) and iHPV Vaccine in Kuwait: Cross-Sectional Survey Study. *JMIR Public Health Surveill* **2025**, *11*, e75818, doi:10.2196/75818.
87. Bashari, N.; Davari, M.; Akbarisari, A.; Khorasani, E.; Daroudi, R. Cost-effectiveness analysis of different types of human papillomavirus vaccination to prevent cervical cancer in Iran. *BMC Public Health* **2025**, *25*, 3927, doi:10.1186/s12889-025-24916-1.
88. Onasoga, O.A.; Dosumu, T.O.; Folarin, A.O.; Shittu, B.M. Knowledge and Uptake of Human Papilloma Virus Vaccine among Female Undergraduate Students in North-Central, Nigeria: A Cross-Sectional Study. *Int J Community Based Nurs Midwifery* **2025**, *13*, 126-137, doi:10.30476/ijcbnm.2025.102762.2509.
89. Mohamed, N.A.; Abdel-Aziz, H.R.; Elsehrawy, M.G. Cultural Influences: Female Students' Awareness and Perception Regarding Human Papillomavirus Vaccine. *SAGE Open Nurs* **2025**, *11*, 23779608251363840, doi:10.1177/23779608251363840.
90. Neisi, N.; Mousavikish, F.; Bastani, M.N.; Parsanahad, M.; Pirmoradi, R. Impact of High-Risk HPV Infection on PI3K, MALAT1, H19 and LINC00460 Expression in Cervical Cells. *J Cell Mol Med* **2025**, *29*, e70949, doi:10.1111/jcmm.70949.
91. Shabanpour, M.; Jalali-Alhosseini, P.; Shoja, Z.; Ghafoori-Ghahdarijani, F.; Taherkhani, S.; Jalilvand, S. Lineage and sublineage analysis of human papillomavirus type 58 in iranian women. *Virol J* **2024**, *21*, 244, doi:10.1186/s12985-024-02521-9.
92. Mobini Kesheh, M.; Barazandeh, M.; Kaffashi, A.; Shahkarami, M.K.; Nadji, S.A. Genetic Diversity of HPV 16 and HPV 18 Based on Partial Long Control Region in Iranian Women. *Can J Infect Dis Med Microbiol* **2022**, *2022*, 4759871, doi:10.1155/2022/4759871.
93. Sadat Larijani, M.; Omrani, M.D.; Soleimani, R.; Bavand, A.; Nejadeh, A.H.; Ezzatizadeh, V.; Jamshidi, M.; Ramezani, A. Determination of Human Papillomavirus Type 18 Lineage of E6: A Population Study from Iran. *Biomed Res Int* **2022**, *2022*, 2839708, doi:10.1155/2022/2839708.
94. Jalali-Alhosseini, P.; Shoja, Z.; Jalilvand, S. Variant analysis of human papillomavirus type 52 in Iranian women during 2018-2020: A case-control study. *Health Sci Rep* **2024**, *7*, e2158, doi:10.1002/hsr2.2158.
95. Taherkhani, S.; Shabanpour, M.; Shoja, Z.; Jalilvand, S. Lineage and Sublineage Analysis of Human Papillomavirus Types 66 and 68 in Iran During 2021-2023: A Cross-Sectional Study. *Health Sci Rep* **2025**, *8*, e70921, doi:10.1002/hsr2.70921.
96. Al-Awadhi, R.; Al-Mutairi, N.; Chehadeh, W. Prevalence of HPV Genotypes in Adult Male Patients with Cutaneous Warts: A Cross-Sectional Study. *Med Princ Pract* **2020**, *29*, 458-464, doi:10.1159/000505599.
97. Köksal, M.O.; Yalçın, B.K.; Keskin, F.; Çiftçi, S.; Yağcı, I.; Hasçiçek, S.; Başaran, B.; Değer, K.; Ağaçfidan, A.; Quaas, A.; et al. Genotype Distribution and Prevalence of Human Papillomavirus in Head and Neck Cancer Samples from Istanbul, Turkey. *Pathogens* **2021**, *10*, doi:10.3390/pathogens10121533.
98. Elhamady, M.M.; Atallah, R.B.; Hashem, O.A.; Abdelaty, M.A.; Elsaie, M.L. Sexual dysfunction and quality of life in males with genital warts. *Basic Clin Androl* **2025**, *35*, 25, doi:10.1186/s12610-025-00274-1.
99. Morhason-Bello, I.; Bello, Y.; Oke, D.; Daniel, A.; Adisa, A.; Fowotade, A.; Zheng, Y.; Akinyemi, J.; Adewole, I.; Pavon, M.A.; et al. Sex differences in the prevalence of high-risk oral and anal human papillomavirus infections among heterosexually active populations in Ibadan, Nigeria. *Front Reprod Health* **2025**, *7*, 1570984, doi:10.3389/frph.2025.1570984.

100. El-Sheikh, N.; Mousa, N.O.; Tawfeik, A.M.; Saleh, A.M.; Elshikh, I.; Deyab, M.; Ragheb, F.; Moneer, M.M.; Kawashti, A.; Osman, A.; et al. Assessment of Human Papillomavirus Infection and Risk Factors in Egyptian Women With Breast Cancer. *Breast Cancer (Auckl)* **2021**, *15*, 1178223421996279, doi:10.1177/1178223421996279.
101. Ceasar, J.; Frank, E.M.; Hall, R.; Hartert, C.; Onks, C. Alternative Allied Health Care Providers: Athletic Trainers Educating Stakeholders to Improve HPV-Related Infections. *J Prim Care Community Health* **2025**, *16*, 21501319251351080, doi:10.1177/21501319251351080.
102. Kottaridi, C.; Resta, P.; Leventakou, D.; Gioti, K.; Zygouras, I.; Gouloumi, A.R.; Sakagiannis, G.; Alzahrani, K.J.; Venetikou, M.S.; Anthouli-Anagnostopoulou, F.; et al. The T350G Variation of Human Papillomavirus 16 E6 Gene Prevails in Oropharyngeal Cancer from a Small Cohort of Greek Patients. *Viruses* **2022**, *14*, doi:10.3390/v14081724.
103. Yahyapour, Y.; Rahmani, R.; Alipour, M.; Alizadeh, A.; Khademian, A.; Sadeghi, F. Prevalence and association of human papillomavirus, Epstein-Barr virus and Merkel Cell polyomavirus with neoplastic esophageal lesions in northern Iran. *Caspian J Intern Med* **2018**, *9*, 353-360, doi:10.22088/cjim.9.4.353.
104. Fakhraei, F.; Haghshenas, M.R.; Hosseini, V.; Rafiei, A.; Naghshvar, F.; Alizadeh-Navaei, R. Detection of human papillomavirus DNA in gastric carcinoma specimens in a high-risk region of Iran. *Biomed Rep* **2016**, *5*, 371-375, doi:10.3892/br.2016.728.
105. Elagali, A.M.; Suliman, A.A.; Altayeb, M.; Dannoun, A.I.; Parine, N.R.; Sakr, H.I.; Suliman, H.S.; Motawee, M.E. Human papillomavirus, gene mutation and estrogen and progesterone receptors in breast cancer: a cross-sectional study. *Pan Afr Med J* **2021**, *38*, 43, doi:10.11604/pamj.2021.38.43.22013.
106. Archin Dialameh, P.; Saki, F.; Monabbati, A.; Dehghanian, A.; Valibeigi, B.; Soveid, M. Detection of Human Papillomavirus in Papillary Thyroid Carcinoma and its Association with Tumor Staging and Pathologic Features. *Iran J Med Sci* **2021**, *46*, 256-262, doi:10.30476/ijms.2020.83135.1191.
107. Alsbeih, G.; Al-Harbi, N.; Bin Judia, S.; Al-Qahtani, W.; Khoja, H.; El-Sebaie, M.; Tulbah, A. Prevalence of Human Papillomavirus (HPV) Infection and the Association with Survival in Saudi Patients with Head and Neck Squamous Cell Carcinoma. *Cancers (Basel)* **2019**, *11*, doi:10.3390/cancers11060820.
108. Fernandes, Q.; Allouch, S.; Gupta, I.; Elmakaty, I.; Elzawawi, K.E.; Amarah, A.; Al-Thawadi, H.; Al-Farsi, H.; Vranic, S.; Al Moustafa, A.E. Human Papillomaviruses-Related Cancers: An Update on the Presence and Prevention Strategies in the Middle East and North African Regions. *Pathogens* **2022**, *11*, doi:10.3390/pathogens11111380.
109. Salhi, I.; Alzahayqa, M.; Qrei, W.; Qawasmi, T.; Qadi, M.; Salah, Z. Genotypic spectrum and prevalence of HPV associated with cervical cancer in Palestine. *BMC Infect Dis* **2025**, *26*, 16, doi:10.1186/s12879-025-12276-5.
110. Keikha, L.; Shahraki-Mohammadi, A.; Esmaeilpour, M. Study of attitudes and knowledge toward human papilloma virus of women aged 15-26 years in Zahedan City in 2024. *J Educ Health Promot* **2025**, *14*, 260, doi:10.4103/jehp.jehp_312_24.
111. Mabrouk Nouh, F.; Abualruz, H.; Al-Zoubi, M.; Al-Mugheed, K.; Ahmed Shahin, H.E.; Ezzat El-Gobashy, R.; Sh Abdullah, A.I.; Alasser, A.M.F.; Al Hrinat, J.; Ghaleb Hendi, A.; et al. Nursing students' knowledge and attitudes toward human papillomavirus vaccination and infection. *Hum Vaccin Immunother* **2026**, *22*, 2609329, doi:10.1080/21645515.2025.2609329.

112. Haddadi, M.; Golestani, A.; Farzi, Y.; Rezaei, N.; Ghasemi, E.; Azadnajafabad, S.; Rezaei, N.; Ahmadi, N.; Rashidi, M.M.; Nasserinejad, M.; et al. Iran is falling behind WHO cervical cancer elimination targets: HPV vaccination coverage and cervical cancer screening participation in 2021. *PLoS One* **2026**, *21*, e0341888, doi:10.1371/journal.pone.0341888.

113. Akhtar, S.; Rezqalla, J.; Alshatti, M.; Ibraheem, A.; Omar, D.; Al-Failakawi, H.; AlHaqqan, S.; AlGhurair, S. Generalized structural equation modeling of demographic disparities in human papillomavirus (HPV) vaccine uptake among female schoolteachers in a middle eastern country. *Infect Agent Cancer* **2026**, *21*, 6, doi:10.1186/s13027-025-00728-1.

114. Hossein Zadeh, S.; Habibnia, S.; Rokni, M. Prevalence and genotype distribution of human papillomavirus (HPV) in an at-risk and suspected population in Iran. *BMC Infect Dis* **2026**, doi:10.1186/s12879-026-13482-5.

115. Finney, D.; Hashim, A.L.O.; Bylappa, S.K.; Mugar, A.E.; Nandhagopal, M.; Jabbar, M.A.; Rahamathullah, N. Genotypic distribution and molecular epidemiology of HPV in women in the UAE using PNA-based RT PCR. *PLoS One* **2026**, *21*, e0346052, doi:10.1371/journal.pone.0346052.

116. Cassani, C.; Dominoni, M.; Pasquali, M.F.; Gardella, B.; Spinillo, A. Multiple human papillomavirus infection and high-grade cervical squamous intraepithelial lesions among women with human immunodeficiency virus: a systematic review and a meta-analysis. *Frontiers in Medicine* **2024**, *Volume 11 - 2024*, doi:10.3389/fmed.2024.1403548.

117. Nikoo, H.R.; Kamali, S.D.; Lashkari, S.; Rezaie, R.; Tabarraei, A.; Yami, H.A.; Joshaghani, H.R.; Mehravar, F.; Azadehrah, M. Epidemiology and genotype distribution of human papillomavirus (HPV) in Northern Iran. *BMC Infect Dis* **2026**, *26*, doi:10.1186/s12879-026-12847-0.

118. Habibpour, H.; Omidvar, S.; Bakhtiari, A.; Hajian-Tilaki, K.; Ghorbani, H. The effect of human genital warts virus on the quality of women's sexual life: A look at the effective factors. *J Educ Health Promot* **2026**, *15*, 122, doi:10.4103/jehp.jehp_1935_24.

119. Chellat, F.Z.; Bahra, N.; Chami Khazraji, Y.; Bekkali, R.; Bennani, M.; Gaizi, Z.; Tachfouti, N.; El Fakir, S.; Ben Abdallah, A.; El Mzibri, M.; et al. HPV Self-Sampling for Cervical Cancer Screening in Morocco: A Cross-Sectional Study. *Asian Pac J Cancer Prev* **2026**, *27*, 1249-1256, doi:10.31557/apjcp.2026.27.4.1249.

120. Karami, A.; Rastin, A.; Shahani, T.; Kazemi Robati, A.; Ahmadi, F.; Jozi, M. Determination of Human Papillomavirus prevalence in the female population from Zanjan, Iran. *Prev Med Rep* **2026**, *62*, 103406, doi:10.1016/j.pmedr.2026.103406.

121. Eslamloueian, A.; Ghaemmaghami, P.; Ghahartars, M.; Janghorban, R. Comparison of sexual distress and sexual avoidance in women with and without genital warts: a case-control study. *BMC Womens Health* **2026**, *26*, doi:10.1186/s12905-026-04263-y.

122. Karami, H.; Soleimani-Jelodar, R.; Khezeli, Z.; Shoja, Z.; Arashkia, A.; Varmazyar, S.; Jalilvand, S. Exploring the association between the lineages of human papillomavirus type 16 and viral physical status in the development of cervical cancer in Iran. *Sci Rep* **2025**, *15*, 43922, doi:10.1038/s41598-025-27658-7.
